# A ketogenic diet can mitigate SARS-CoV-2 induced systemic reprogramming and inflammation

**DOI:** 10.1038/s42003-023-05478-7

**Published:** 2023-11-03

**Authors:** Amelia Palermo, Shen Li, Johanna ten Hoeve, Akshay Chellappa, Alexandra Morris, Barbara Dillon, Feiyang Ma, Yijie Wang, Edward Cao, Byourak Shabane, Rebeca Acín-Perez, Anton Petcherski, A. Jake Lusis, Stanley Hazen, Orian S. Shirihai, Matteo Pellegrini, Vaithilingaraja Arumugaswami, Thomas G. Graeber, Arjun Deb

**Affiliations:** 1 Department of Molecular and Medical Pharmacology, David Geffen School of Medicine, University of California, Los Angeles, CA 90095 USA; 2 California Nanosystems Institute, University of California, Los Angeles, CA 90095 USA; 3 UCLA Metabolomics Center, University of California, Los Angeles, CA 90095 USA; 4 Crump Institute for Molecular Imaging, University of California, Los Angeles, CA 90095 USA; 5 Division of Cardiology, Department of Medicine, David Geffen School of Medicine, University of California, Los Angeles, CA 90095 USA; 6 UCLA Cardiovascular Research Theme, David Geffen School of Medicine, University of California, Los Angeles, CA 90095 USA; 7 Department of Molecular, Cell and Developmental Biology, Division of Life Sciences, University of California, Los Angeles, CA 90095 USA; 8 Eli & Edythe Broad Center of Regenerative Medicine and Stem Cell Research, University of California, Los Angeles, CA 90095 USA; 9 Molecular Biology Institute, University of California, Los Angeles, CA 90095 USA; 10 Department of Genetics, David Geffen School of Medicine, Los Angeles, CA 90095 USA; 11 Department of Environment, Health and Safety, University of California, Los Angeles, CA 90095 USA; 12 Division of Rheumatology, Department of Internal Medicine, University of Michigan, Ann Arbor, Michigan 48109 USA; 13 Department of Medicine, Endocrinology, David Geffen School of Medicine, University of California, Los Angeles, CA 90095 USA; 14 Department of Cardiovascular and Metabolic Sciences, Cleveland Clinic, Cleveland, OH 44195 USA

**Keywords:** Metabolomics, Viral infection

## Abstract

The ketogenic diet (KD) has demonstrated benefits in numerous clinical studies and animal models of disease in modulating the immune response and promoting a systemic anti-inflammatory state. Here we investigate the effects of a KD on systemic toxicity in mice following SARS-CoV-2 infection. Our data indicate that under KD, SARS-CoV-2 reduces weight loss with overall improved animal survival. Muted multi-organ transcriptional reprogramming and metabolism rewiring suggest that a KD initiates and mitigates systemic changes induced by the virus. We observed reduced metalloproteases and increased inflammatory homeostatic protein transcription in the heart, with decreased serum pro-inflammatory cytokines (*i.e*., TNF-α, IL-15, IL-22, G-CSF, M-CSF, MCP-1), metabolic markers of inflammation (*i.e*., kynurenine/tryptophane ratio), and inflammatory prostaglandins, indicative of reduced systemic inflammation in animals infected under a KD. Taken together, these data suggest that a KD can alter the transcriptional and metabolic response in animals following SARS-CoV-2 infection with improved mice health, reduced inflammation, and restored amino acid, nucleotide, lipid, and energy currency metabolism.

## Abstract (web summary)

A systems-wide study of mice infected with SARS-CoV2 under ketogenic diet shows that this diet reprograms the transcriptional and metabolic response in animals following infection with improved health, reduced inflammation, and restored metabolism.

## Introduction

Severe acute respiratory syndrome coronavirus 2 (SARS-CoV-2), the cause of coronavirus disease 2019 (COVID-19), has irreversibly impacted human health and life expectancy^1^. COVID-19 can lead to heterogeneous symptoms, which span from asymptomatic infections to respiratory failure with systemic toxicity and multi-organ dysfunction^2–4^. Observational studies of COVID-19 revealed that metabolic pro-inflammatory comorbidities such as obesity and diabetes, are associated with disease severity^5,6^. The interplay between metabolism and the immune-inflammatory response can significantly impact patient health during viral infection^7–12^. In particular, the implementation of a ketogenic diet (KD) can be harnessed to improve immunity and protect from inflammatory damage^13^. The KD regimen has shown effective in enhancing immunity across independent studies and in activating protective γδ T cells^14–16^. Consequently, systemic reprogramming induced by the KD may alter COVID-19 multi-organ toxicity^17,18^.

The ketogenic diet (KD) entails the intake of high-fat, low-carbohydrate foods that activate the production of ketone bodies *i.e*., β-hydroxybutyrate (BHB), acetoacetate (ACA) and acetone (AC)^19^. In ketogenesis, lipids and proteins are used to support cellular energy demands by providing metabolic intermediates (ketone bodies) that function as fuel in lieu of glucose^20,21^. Similarly to starvation and fasting, under KD peripheral fat is mobilized towards the liver for ketogenic catabolism of fatty acids *via* β-oxidation to acetyl-CoA, later converted in BHB, ACA, AC. These are distributed to the peripheral tissues and central organs to sustain energy demands while bypassing glucose oxidation (Supplementary Fig. 1)^22^.

In our recent study, we demonstrated that SARS-CoV-2 induces severe systemic extrapulmonary toxicity and metabolic reprogramming of vital organs in a murine model within 7 days of infection^23^. Mice expressing the hACE2 transgene *via* adenoviral delivery and infected with SARS-CoV-2 showed suppression of oxidative phosphorylation (OXPHOS) and of the tricarboxylic acid cycle (TCA) at the transcriptional level, accompanied by severe myocardial toxicity (decreased heart rate, myocardial edema, and myofibrillar disarray). Key metabolites of the TCA cycle showed directional decrease consistent with the transcriptional response^23^. This phenotype was associated with profound weight loss, massive peripheral fat mobilization, and morbidity within 7 days from infection, which led us to hypothesize that ketogenesis may affect the systemic response to SARS-CoV-2.

**Fig. 1:**
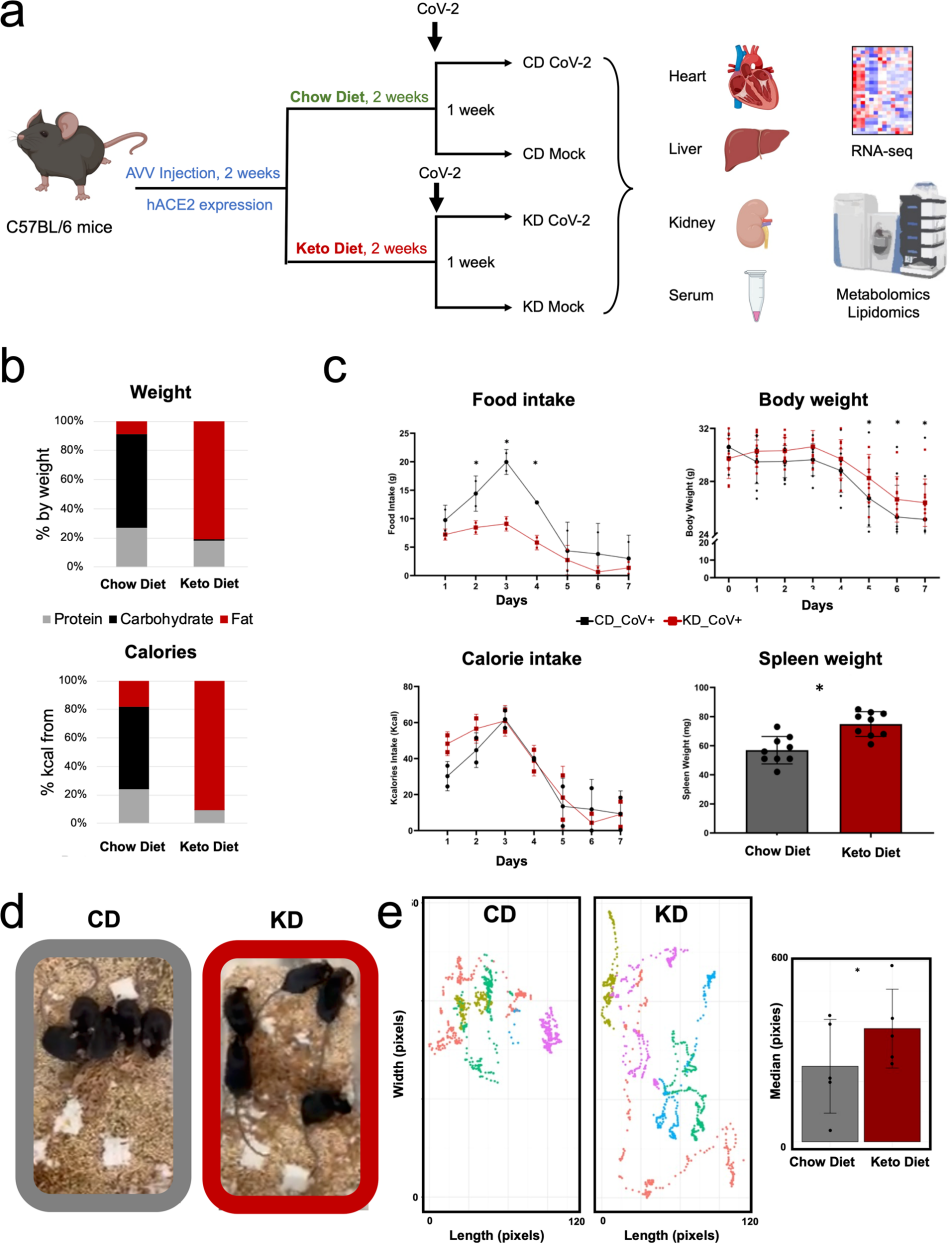
A ketogenic diet improves mice health and rescues mice behavior in SARS-CoV2 infection. **a** Schematization of the study; (**b**) KD and CD composition by weight and by calories; (**c**) effect of a KD on mice food and calorie intake (data obtained as average of mice cage consumption, cage *n* = 2), body and spleen weight over 7 days after infection in C57Bl/6 male mice (14–17 weeks) (* significance *p* threshold 0.05, *n* = 5 for body weight and *n* = 9 for spleen weight biologically independent samples); (**d**) a KD rescues mice mobility and behavior after 7 days from infection (*n* = 5 biologically independent samples); (**e**) distance traveled by each mouse over 17 s (in pixels, colors represents different mice) (right) and average distance traveled by infected mice under CD or KD (*significance *p* threshold 0.05). (Created with BioRender.com).

Here we sought to determine whether a KD may improve mice health by reducing extrapulmonary systemic toxicity and inflammation induced by infection with SARS-CoV-2. First, we developed a murine model susceptible to viral infection that recapitulates metabolic and transcriptional changes of systemic ketosis. Next, we monitored mice health over one week from infection under KD or control (chow) diet (CD) and performed multi-organ transcriptional and metabolic profiling (Fig. 1a). Our data indicate that a KD attenuates systemic toxicity following SARS-CoV-2 infection. Reduced multi-organ transcriptional reprogramming suggests that a KD anticipates adaptive systemic changes induced by viral infection. We observed reduced metalloproteases and increased inflammatory homeostatic gene transcription in the heart and liver. Further analysis performed on mice serum revealed decreased pro-inflammatory cytokines (*i.e*., TNF-α, IL-15, IL-22, G-CSF, M-CSF, MCP-1), metabolic markers of inflammation (*i.e*., kynurenine/tryptophane ratio), and pro-inflammatory prostaglandins, indicative of reduced systemic inflammation in animals infected under KD. Metabolomics profiling also indicated decreased metabolism rewiring in the heart and rescued amino acid, nucleotide, energy currency metabolites, acyl-Coenzyme A pool (acyl-CoAs), and lipid precursors in all tissues of KD fed mice. These changes were accompanied by a restoration of mitochondrial fuel utilization driven by changes in complex I/II ratios and respirasome assembly.

This study demonstrates that a KD can mitigate transcriptional reprogramming following SARS-CoV-2 infection in mice, and leads to reduced systemic toxicity/inflammation, rescued metabolic abnormalities, and significantly better survival. Taken together our observations provide a solid rationale to investigate the efficacy of targeted dietary and metabolic interventions for improved COVID-19 acute and potentially chronic (long COVID) disease outcomes.

## Results

### A KD attenuates systemic toxicity in animals following SARS-CoV-2 infection

We set out to determine whether a KD may mitigate SARS-CoV-2 induced systemic toxicity and overall animal health. We used a murine model of SARS-CoV-2 systemic toxicity we have recently described^23^. In this model, C57Bl/6 mice are injected with adeno-associated vectors AAV-9-hACE2 or AAV-9 GFP, followed by injection of vehicle or live SARS-CoV-2 virus. To determine the role of ketogenesis in affecting systemic toxicity, animals were placed on a KD or CD *ad libitum* for 2 weeks. CD and KD composition by weight and energy values (kcal) are reported in Fig. 1b. Briefly, the control group was fed a fixed formula 18% protein rodent diet (Teklad Global 18% Protein Rodent Diet, Envigo), the KD group was exposed to a purified formula 16% protein and 67% in vegetable oils with trans fatty acids for a ratio of fat to protein of 4.2 (Teklad Ketogenic Rodent Diet TD. 190049, Envigo).

Serum BHB increased after two weeks of KD in hACE2-AAV-9 mice, while glucose levels remained unchanged (Supplementary Fig. 2a). Prior to SARS-CoV-2 infection, both mice on CD and KD maintained steady body weight (Supplementary Fig. 2b). RNA-seq gene expression analysis of tissues harvested after 2 weeks of CD or KD (*n* = 3/group biologically independent samples) showed distinct expression patterns in the liver as opposed to the kidney and heart, as shown by principal component analysis (PCA), PC1 (PC1: 53% total explained variance) (Supplementary Fig. 3a). Genes involved in ketogenesis, lipid β-oxidation and other PPARα targets were upregulated in the liver, while genes involved in lipid synthesis (Fas, Scd-1, Srebp-1c) were suppressed (Supplementary Fig. 3b). Gene Ontology (GO) process pathway enrichment analysis of differentially regulated genes (DEGs) confirmed the upregulation of lipid catabolism, β-oxidation, and oxygen transport (Supplementary Fig. 3c). These data demonstrate robust ketogenesis in animals following 2 week administration of KD versus CD.

Next, we infected AAV-9-hACE2 mice (*n* = 5/group biologically independent samples) with SARS-CoV-2 after two weeks of exposure to a CD or KD diet (named “CD SARS-CoV-2” and “KD SARS-CoV-2” groups, respectively). Uninfected AAV-9-hACE2/vehicle mice under CD or KD diet were used as control (named “CD” and “KD” groups, respectively).

Mice on a KD showed reduced food intake but similar calorie intake over one week from infection, an observation consistent with the different caloric content of the CD and KD pellets (6.7 kcal/g for KD, 3.1 kcal/g for CD pellets). With the progression of time from infection, both CD and KD groups showed a significant decrease in food and calorie intake. However, the implementation of a KD significantly reduced mice body weight loss and spleen weight loss at day 5, 6 and 7 from infection (Fig. 1c). This observation was in contrast to SARS-CoV-2 induced body weight loss and spleen atrophy/reduction recorded in our previous study^23^. No significant changes were observed in serum glucose and BHB levels (Supplementary Fig. 4). Over 7 days from infection, animals under a CD showed profound morbidity and severely restricted activity defined by limited mobility and lethargic behavior, consistent with our previous report^23^. Animals infected under KD demonstrated rescued behavior with normal activity and mobility (Fig. 1d, e, Supplementary Video 1 and 2**)**. These data demonstrate that the KD is beneficial to mice overall health and behavior following SARS-CoV-2 infection.

### A KD anticipates SARS-CoV-2 induced systemic transcriptional reprogramming

The KD broadly reprograms gene expression at system-level^24^. In our previous report, we showed that SARS-CoV-2 induces profound multi-organ transcriptional changes^23^. Therefore, we established whether a KD may affect SARS-CoV-2 induced systemic reprogramming at the transcriptional level by examining gene expression changes in extrapulmonary organs.

We performed multi-organ RNA-seq of mice tissues (*n* = 3/group biologically independent samples) after two weeks of exposure to a CD or KD and after one week from infection. Uninfected AAV-9/hACE2 + /vehicle mice under CD or KD diet regimen served as control (Fig. 1a).

**Fig. 2:**
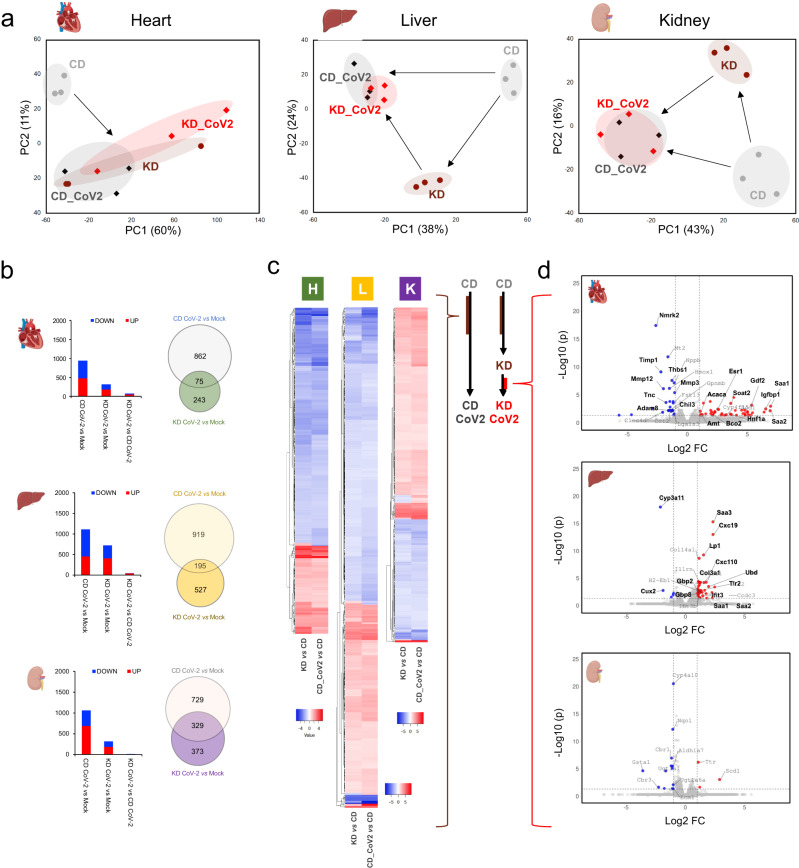
A ketogenic diet anticipates SARS-CoV-2 induced systemic reprogramming and affects matrix remodeling and inflammatory homeostasis at the transcriptional level. **a** Principal component analysis of differentially expressed genes (DEGs) in the heart, liver, and kidney indicates that a KD shifts transcriptional profiles towards SARS-CoV2 induced changes; (**b**) number of significantly up and down-regulated genes in each tissue and Ven diagrams showing the number of unique and shared genes dysregulated in SARS-CoV2 infection under CD or KD diet in distinct tissues; (**c**) heatmap showing log2FC values for DEGs consistently induced by the KD and by viral infection in the control group (CD); (**d**) volcano plots showing DEGs in KD infected vs CD infected mice (significantly down-regulated genes in blue, up-regulated genes in red); (adjp <0.05 and log2 FC > |1| for all panels, dotted lines indicate significance and FC thresholds, *n* = 3/group biologically independent samples). (Created with BioRender.com).

PCA demonstrated distinct transcriptional patterns in uninfected and infected animals under KD and CD, with PC1 explaining 60%, 38%, and 43% of total variability between groups, in the heart, liver, and kidney, respectively. Projected PC1 and PC2 distances suggested that the KD induces transcriptional changes in part similar to those caused by viral infection in all tissues (Fig. 2a).

Paired analysis showed ~66% (619 genes), ~35% (392 genes) and ~70% (741 genes) decrease in DEGs (adjp < 0.05, log2FC > 1) in the KD infected group as opposed to CD infected animals in the heart, liver, and kidney, respectively (Fig. 2b). DEGs between infected KD and infected CD mice were 74, 45 and 16 in heart, liver, and kidney, respectively, demonstrating similar overall transcriptional programs. These data corroborate the hypothesis that a KD shifts the transcriptional baseline of uninfected mice towards changes induced by SARS-CoV-2.

Next, we compared DEGs due to the exposure to a KD in uninfected animals (KD *vs* CD), with those changing because of SARS-CoV-2 infection under CD (CD-SARS-CoV-2 *vs* CD) (Fig. 2c). This analysis showed that 327 (heart), 499 (liver), and 341 (kidney) of the genes reprogrammed by the KD were also affected during SARS-CoV-2 infection under CD. Of these, approximately 99% showed consistent up or down regulation in all tissues, indicating that 35% (heart), 45% (liver) and 32% (kidney) of transcriptional changes induced by SARS-CoV-2 in CD can be anticipated/primed through the implementation of a KD. GO process enrichment analysis of shared DEGs pointed to lipid and acetyl-CoA metabolism reprogramming across all tissues with regulation of PPARα targets, such as *Hmgcs2*, *Cidea*, *Cidec*, *Ehhadh*, *Angptl4*, *Ucp3*, *Acot1-4*, *Slc27a1* and *Fabp2* (Supplementary Data 1 and 2, Supplementary Fig. 5a). In the liver, *Acaca* and *Acsl6* were decreased, and *Cyp7a1*, *Cyp8b1*, *Cyp2b9* (steroid/cholesterol metabolism) were upregulated. RNA-seq data also showed increased CPT1A in the liver and kidney (Supplementary Fig. 5a).

Reversed-phase liquid chromatography (RP-LC) and untargeted mass spectrometry (MS) of polar lipids in SARS-CoV-2 *vs* control tissues under CD confirmed increased medium and long chain acyl-carnitines in the liver (Supplementary Fig. 5b). Transcription factor regulatory network analysis of RNA-seq data by the transcriptional factor (TF)-target interaction database Transcriptional Regulatory Relationships Unraveled by Sentence-based Text mining (TRRUST, v2) predicted 33, 64, and 49 TFs in heart, liver, and kidney, respectively^25,26^. PPARα was consistently predicted among the top 5 most significant TFs (Supplementary Fig. 5c). Serum levels of BHB showed heterogeneous patterns in infected mice (Supplementary Fig. 5d).

This evidence supports the finding that ketogenesis is activated at the transcriptional level in extrapulmonary tissues following SARS-CoV-2 infection. Consistently, we previously observed peripheral fat mobilization, loss of adipose tissue, and reduced adipocyte size in the same SARS-CoV-2 murine model of infection^23^.

Taken together, these data demonstrate a systemic transcriptional switch towards ketogenic metabolism after SARS-CoV-2 infection under CD and support the hypothesis that the implementation of a KD prior to infection primes the system by anticipating transcriptional adaptations induced by the virus.

### A KD affects matrix remodeling and inflammatory response at the transcriptional level in SARS-CoV-2

We compared transcriptional changes in animals at the endpoint of our study, i.e. mice infected under KD *vs* CD (KD-SARS-CoV-2 *vs* CD-SARS-CoV-2). The endpoint of 7 days was primarily determined by the moribund health state of the CD SARS-CoV-2 infected animals that required euthanasia. This analysis detected 74 DEGs in the heart (26 down-, 48 up-regulated), 45 in the liver (6 down-, 39 up-regulated), and 16 in the kidney (13 down-, 3 up-regulated) (Fig. 2d, Supplementary Data 4).

In the heart, *Nmrk2* was significantly downregulated in the KD-SARS-CoV-2 group. This gene is a minimally expressed nicotinamide riboside kinase isoform of *Nmrk1* that has been reported to increase during SARS-CoV-2 infection and is involved in NAD+ synthesis through *salvage* pathways^27^. Decreased *Timp1*, *Thbs1*, *Tnc*, *Adam8*, *Chil3*, *Mmp-12* and *Mmp-3* transcription also suggested reduced matrix remodeling associated with lower inflammation in the KD-SARS-CoV-2 group (Fig. 2d).

On the contrary, serum amyloid A1 and A2 (*Saa1* and *Saa2*), *Igfbp1*, *Gdf2*, *Hnf1a*, *Soat2*, *Bco2*, *Amt*, *Acaca*, and *Esr1* were upregulated in cardiac tissue in the KD infected mice.

In the liver, we detected increased *Saa3*, *Cxc19*, *Lp1*, *Cxc110*, *Ubd*, *Col3a1*, *Tlr2*, *Ifit3*, *Gbp2*, and *Gbp8* in the KD-SARS-CoV-2 group, implicated in interferon response, innate immunity, and collagen remodeling. *Cyp3a11* and *Cux2* were instead reduced. *Saa3* and slightly significant *Saa1* and *Saa2* upregulation in infected KD fed mice is not ascribable to the sole effect of a KD, as shown by the downregulation of *Saa1* and no significant changes for other *Saa2* and *3* in the liver of uninfected mice under KD (Supplementary Data 5). Few significant changes were detected in the kidney (Supplementary Data 4).

Network analysis of GO process enrichment in the heart predicted the downregulation of defense response, eosinophil migration, regulation of immune system process, leukocyte activation, wound healing, and tumor necrosis factor superfamily cytokine production while pointing to increased cholesterol and steroid biosynthesis, and lipid metabolism (Supplementary Fig. 6 and Supplementary Data 6). These data suggest that under KD, SARS-CoV-2 induced matrix remodeling and inflammatory response are mitigated, in concert with reduced NAD+ metabolism dysregulation and induced cellular lipid and steroid metabolism.

### A KD attenuates the cytokine storm and reduces systemic inflammation during infection

**Fig. 3:**
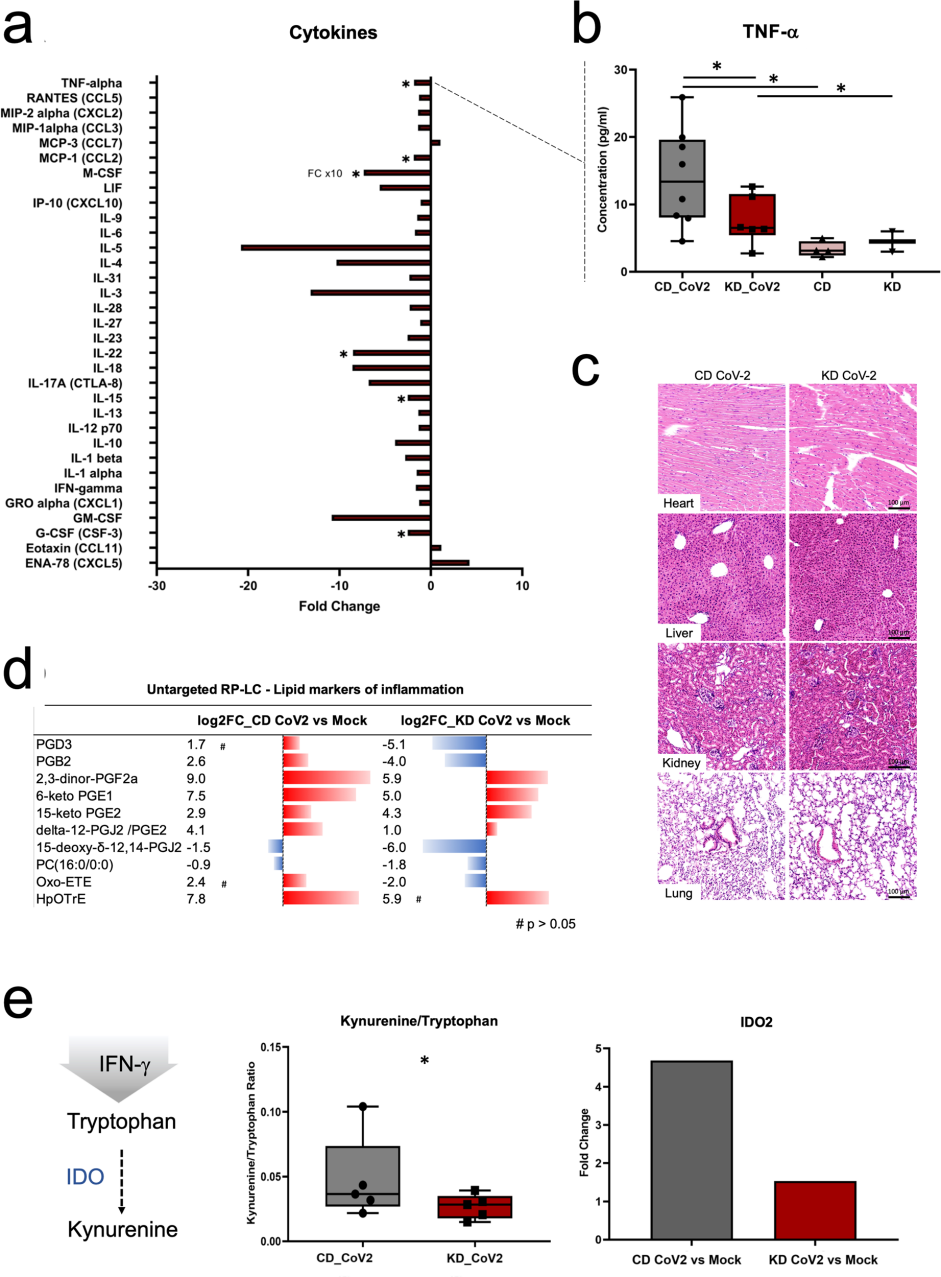
A ketogenic diet reduces markers of acute inflammation in SARS-CoV-2 infection. **a** Fold change of serum cytokines showing significant concentration changes during infection under KD *vs* CD regimen (*n* = 8/group biologically independent samples, * for *p* < 0.05 and fold change > |1 | ); (**b**) serum concentration **(**pg/mL) of tumor necrosis factor-alpha (TNF-a) in CD- and KD-SARS-CoV-2 infected and mock mice; (**c**) Hematoxylin-Eosin staining of heart, liver, kidney, and lung in CD864 and KD-SARS-CoV-2 mice (*n* = 6/group biologically independent samples); (**d**) untargeted RP-LC analysis of serum showing up-regulation of retinoic acid and inflammatory lipid mediators (eicosanoids) in CD-SARS-CoV-2 infected mice, and corresponding decrease under KD regimen (*n* = 5/group biologically independent samples, significance *p* < 0.05, fold change > |2 | , # indicates *p* > 0.05); (**e**) kynurenine/tryptophane ratio metabolic marker of acute inflammation in serum (right) (* for p 0.05, *n* = 5/group biologically independent samples), and SARS-CoV-2 induced upregulation of IDO2 (indoleamine-2,3-dioxygenase) in hearth (* for p 0.05 ang log2FC for gene expression > |1 | , *n* = 3/group biologically independent samples).

To determine whether a KD may alter serum markers of systemic inflammation during SARS-CoV-2 infection, we measured the concentration of pro-inflammatory cytokines in samples from mice infected under CD or KD using the ProcartaPlex 1A Panel that enables the analysis of 36 mouse pro-inflammatory cytokine and chemokines in a single well by Luminex xMAP technology. Our data show that interleukin-15 (IL-15, −2.5 FC), IL-22 (−8.5 FC), macrophage colony stimulating factor (M-CSF, −73.5 FC), granulocyte colony-stimulating factor (G-CSF, −2.4 FC), monocyte chemoattractant protein-1 (MCP-1, −1.9 FC) and tumor necrosis factor-α (TNF-α −1.8 FC) were significantly decreased during infection in mice fed a KD (Fig. 3a, b). Hematoxylin and eosin (HE) tissue staining showed no histological abnormalities or differences between the two groups (Fig. 3c).

Next, we performed untargeted RP LC-MS analysis in electrospray negative and positive ionization modes (ESI + /-) to profile polar lipids in serum samples at day 7 from infection in CD and KD fed mice. We detected a total of ~9500 metabolic features in serum samples analyzed in ESI- mode, and a total of ~14500 metabolic features in ESI+ mode. Of these, 360 (106 down, 254 up) and 110 (82 down, 28 up) metabolic features were significantly dysregulated (*p* < 0.01, FC > 2) in ESI- in CD infected or KD infected *vs* mock mice, respectively. In ESI + , a total of 245 (93 up, 152 down) and 131 (10 up, 122 down) metabolic features were significantly dysregulated in CD-SARS-CoV-2 or KD-SARS-CoV-2 *vs* CD or KD mock mice, respectively. Further accurate *m/z* match with reference lipid repositories and *MS*/MS fragmentation pattern analysis led to the characterization of 10 metabolic features (Fig. 3d). These included 8 eicosanoids (prostaglandins) mediators of acute inflammatory response with higher FC in CD *vs* KD infected mice.

Hydrophilic interaction liquid chromatography (HILIC) MS analysis confirmed lower serum levels of the kynurenine/tryptophane ratio, a metabolic index upregulated in inflammatory states. RNA-seq data also showed decreased expression of indoleamine 2,4-dioxysenase 2 (*IDO2*) in the heart in the KD-SARS-CoV-2 group (Fig. 3e), consistent with decreased kynurenine/tryptophan ratios.

Taken together, these data suggest that a KD may be a preventive non-invasive approach to decrease SARS-CoV-2 associated systemic toxicity through reduced inflammation.

### A KD mitigates metabolic changes induced by SARS-CoV-2

We next investigated whether a KD may affect metabolic changes induced by the infection. Metabolite abundances were charted by targeted metabolomics of central carbon metabolism and related pathways by HILIC-MS after metabolite extraction from tissues and serum.

**Fig. 4:**
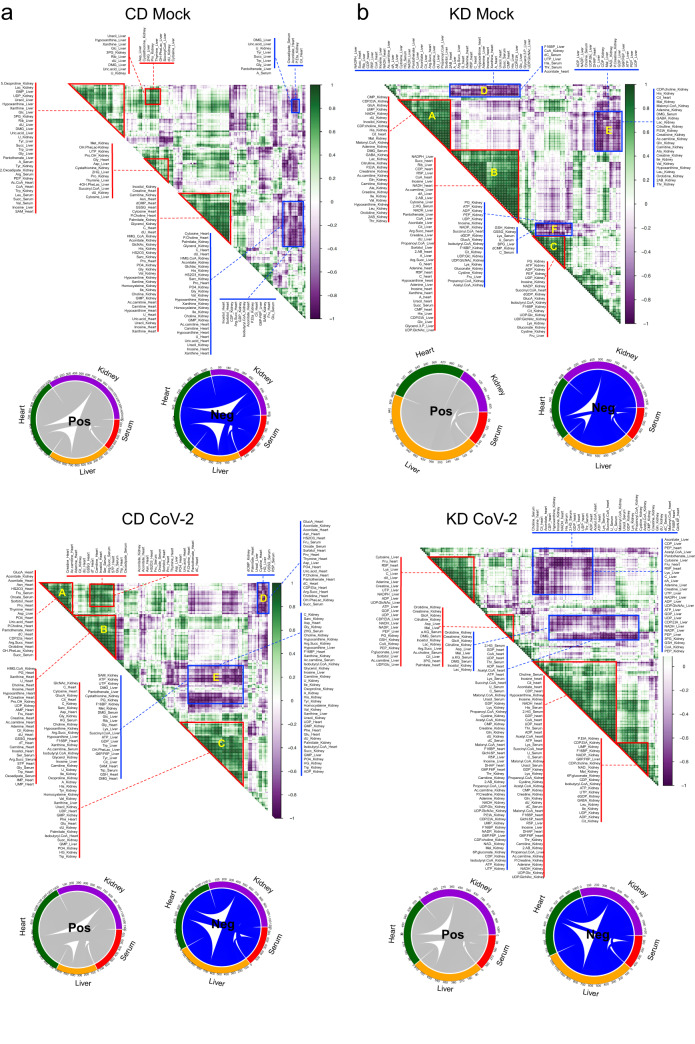
SARS-CoV-2 induces distinct intra- and inter-tissue metabolism reprogramming in CD and KD mice. **a** Metabolite intra- and inter-tissue correlation maps and circos representations of positive (pos, gray) and negative (neg, blue) inter-tissue correlations for CD uninfected (upper panel) and CD-SARS-CoV-2 mice (lower panel) at day 7 from infection; (**b**) metabolite intra- and inter-tissue correlation maps and circos representations of positive (pos, gray) and negative (neg, blue) inter-tissue correlations for KD uninfected (upper panel) and KD-SARS-CoV-2 mice (lower panel) at day 7 from infection (average hierarchical clustering of Pearson correlation matrix, red triangles/boxes highlight highly significant correlations, i.e. *p* < 0.05, *n* = 5/group biologically independent samples).

To determine the effect of a KD on metabolite intra- and inter-tissue associations, we computed Pearson correlation coefficients for the CD and KD, CD-SARS-CoV-2 and KD-SARS-CoV-2 groups, for metabolites with significant changes in paired univariate analysis in at least one tissue (i.e., in KD-SARS-CoV-2 *vs* KD or CD-SARS-CoV-2 *vs* CD) (Fig. 4a, b). As expected, a KD increased positive and negative metabolite intra- and inter-organ correlations (significance *p* < 0.05, Supplementary Data 7), with a higher overall number of positive associations between the liver and the heart and serum, and negative associations with the kidney (Fig. 4b, upper panel). A KD diet also induces stronger positive associations (higher correlation coefficient, lower *p* value) between metabolites in the heart and liver, and within the kidney (Fig. 4b, upper panel, clusters A, B, and C), and negative associations between the kidney and all other tissues (clusters E and F).

In the CD-SARS-CoV-2, viral infection increases negative associations between the liver and the kidney, while leaving unchanged positive associations between the liver and other tissues (Fig. 4a, circos plots). Stronger positive intra-tissue correlations were observed in the heart (Fig. 4a, lower panel, clusters A-C) and kidney (cluster D). In the KD-SARS-CoV-2 group, the infection led to weaker intra-tissue positive associations in the liver and kidney, as opposed to the KD mock group (Supplementary Data 7). Negative associations were also weaker than those observed in the KD mock group (Fig. 4b, lower panel). This suggests that SARS-CoV-2 increases hepatic metabolic intra- and inter-tissue associations, particularly with the heart and kidney, and that a KD can mitigate these changes during infection.

**Fig. 5:**
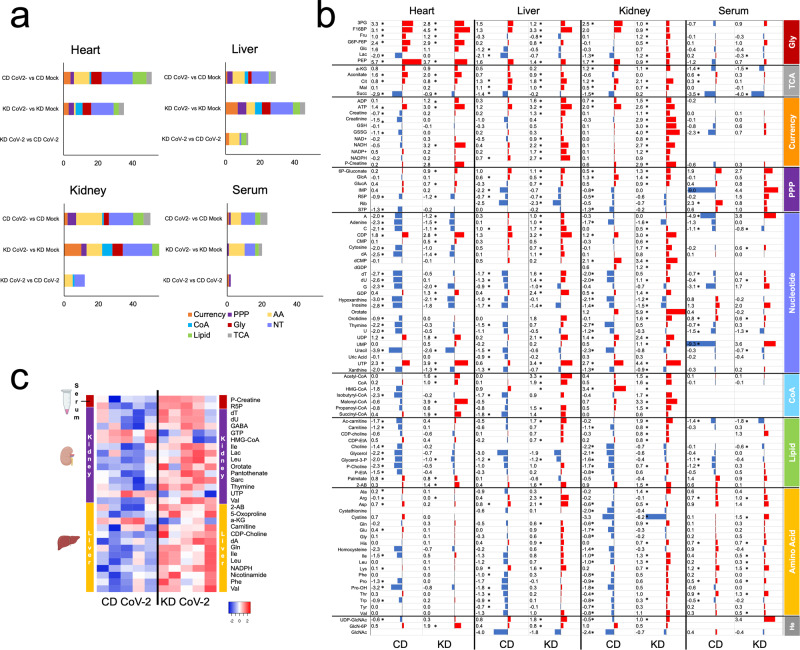
A ketogenic diet counteracts metabolic changes induced by SARS-CoV-2 infection. **a** Biological classification of total altered metabolites in serum, heart, liver and kidney (*n* = 5/group biologically independent samples, *p* < 0.05 and FC > |1.5 | ); (**b**) log2FC of metabolites showing significant changes in paired analysis of CD-SARS-CoV-2 *vs* CD or KD-SARS-CoV-2 *vs* KD (indicated as CD and KD, respectively, *n* = 5/group biologically independent samples, *p* < 0.05 and FC > |1 | ); (**c**) heatmap of log2 intensities for significant metabolites in KD-SARS-CoV-2 *vs* CD-SARS-CoV-2 mice for serum, kidney, and liver (*n* = 5/group) biologically independent samples, *p* < 0.05 and FC > |1 | , no significant features in the heart. Metabolite abbreviations are reported in Supplementary Data 8.

Next, we performed paired univariate analysis of metabolite abundances, i.e. KD-SARS-CoV-2 *vs* KD and CD-SARS-CoV-2 *vs* CD. Infection under KD led to reduced changes in heart and serum, accompanied by increased metabolism reprogramming in the liver and kidney (Fig. 5a). We detected a general increase in currency metabolites, and of acyl-CoAs across all tissues in KD-SARS-CoV-2 mice (Fig. 5b, metabolite abbreviations available in Supplementary Data 8). Metabolites in nucleotide metabolism were increased in the kidney, liver, and serum with a less pronounced effect in the heart. This is particularly evident for A, G and UMP in serum, which showed more than 5 log2FC relative increase. IMP was increased in serum under KD (+4.4 log2FC) while decreased in the CD group (−9 log2FC).

In all tissues, the KD rescues lipid precursors and amino acid levels in infected mice (both depleted in animals infected under CD). Tryptophan levels are unaffected in the KD group, an observation consistent with reduced IDO activity, decreased kynurenine/tryptophan ratio, and inflammation in KD infected mice (Fig. 3).

Univariate analysis of metabolomics data for infected mice under CD or KD at day 7 from infection (i.e., KD-SARS-CoV-2 *vs* CD-SARS-CoV-2), showed no differences in heart and serum, the latter showing increased P-creatine and R5P only. In the kidney and liver, we detected increased nucleotides (dT, dU, orotate, thymine, dA), amino acids (isoleucine, leucine, valine, glycine, phenylalanine) and lipid precursors (HMG-CoA, CDP-choline, carnitine) (Fig. 5c) in the KD infected group. This suggests that a KD primes the myocardium to anticipate metabolic changes induced by the virus by counteracting nucleotide, amino acid and lipid metabolism alterations caused by the infection. PCA of metabolites changing in at least one tissue/condition supports this interpretation (Supplementary Fig. 7).

### A KD rescues Complex I/II respiratory ratios and respiratory supercomplexes (RSC) assembly

**Fig. 6:**
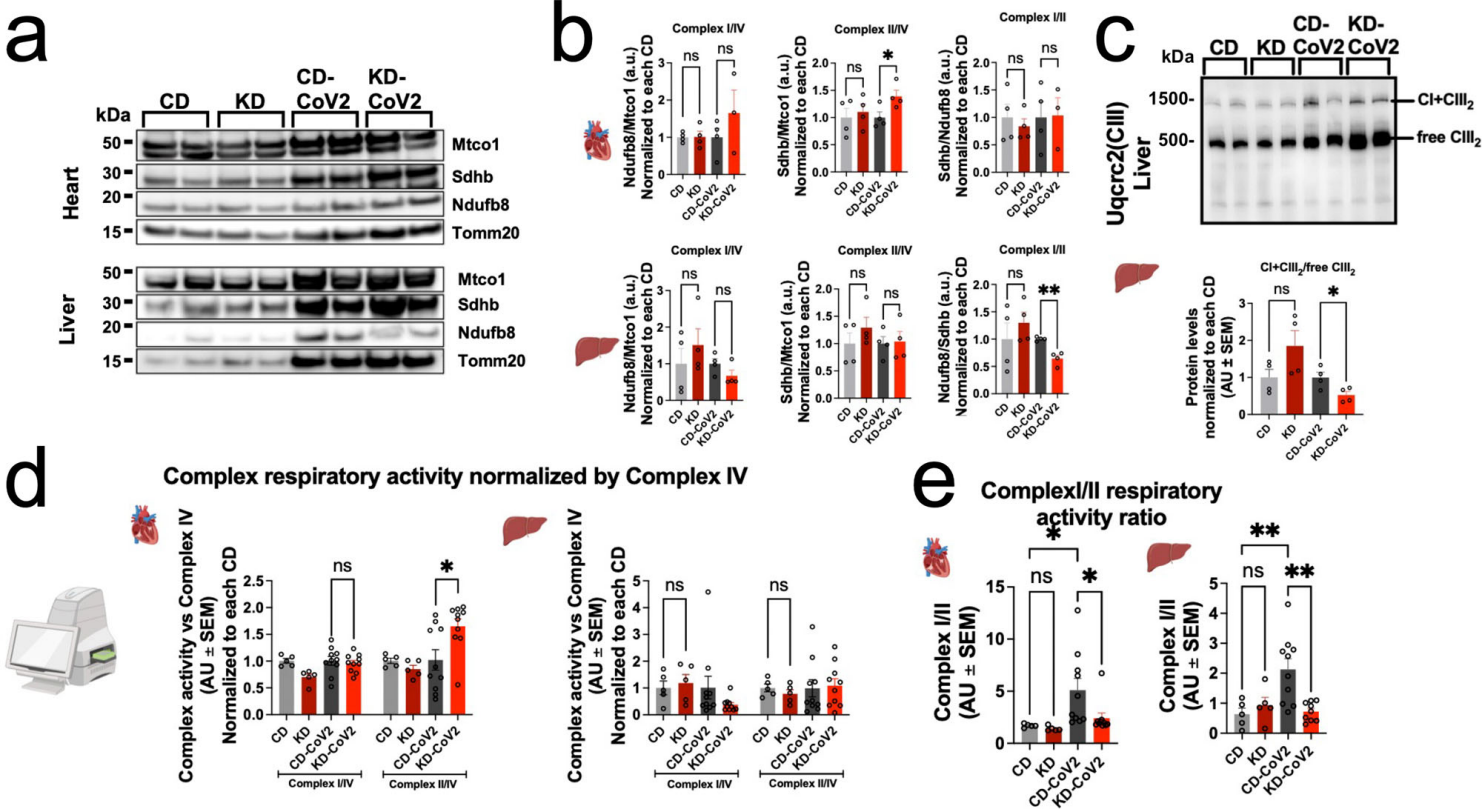
A ketogenic diet rescues Complex I/II respiratory ratios and respiratory supercomplexes assembly. **a** Immunoblots of complex I (Ndufb8), complex II (Sdhb), complex IV (Mtco) subunits, and Tomm20 in heart (top) and liver (bottom). Whole membranes are displayed in Supplementary Fig. 8A; (**b**) Immunoblot quantifications of complex I (left) and II (center) subunit levels normalized by complex IV and ratios of complex I/II levels (right) in the heart (top) and liver (bottom) normalized to each CD condition. Graphs represent mean ± SEM of *n* = 3–4 biologically independent samples; (**c**) Immunoblot of blue native gel electrophoresis stained for Uqcrc2 (top) and quantification of assembled CI + CIII_2_ versus unassembled CIII_2_ respirasome ratios in the liver normalized to each CD condition (bottom). Graphs represent mean ± SEM of *n* = 4 biologically independent samples; (**d**) Respiratory activity of complexes I and II normalized by complex IV and each CD condition in the liver (left) and heart (right) measured by RIFS. Graphs represent mean ± SEM of *N* = 5–10 biologically independent samples; (**e**) respiratory ratios of complex I/II in the heart (left) and liver (right). Graphs represent mean ± SEM of *n* = 5–10 biologically independent samples. Statistical significance for pairwise comparisons in (**b**, **c**, **d**) was determined using unpaired 2-tailed Student’s *t* test or Mann–Whitney test where appropriate. Statistical significance of group comparisons in (**e**) was determined by One-way ANOVA with Holm-Šídák’s multiple comparisons test. * = *p* < 0.05, ** = *p* < 0.01. (Created with BioRender.com).

SARS-CoV-2 induced metabolic reprogramming diverts mitochondrial fuel utilization away from electron transport chain (ETC) complex II driven fatty acid oxidation to glycolytic derived intermediates^28^. Since a KD can enhance complex II activity^29^, we analyzed levels of respiratory complexes by immunoblot, RSC by blue native electrophoresis (BN-PAGE), and respiratory complex activity using respirometry in frozen samples (RIFS)^29^. The KD per se did not alter levels of respiratory complex I (CI) and complex II (CII) relative to complex IV (CIV) in heart and liver tissues (Fig. 6a, b); however, during SARS-CoV-2 infection, the KD increased relative CII levels in the heart and trended to decrease relative CI in the liver by 50% (*p* = 0.2) (Fig. 6a, b). This trend led to a significant change in CI/CII level ratio in the liver (Fig. 6b).

Analysis of mitochondrial mass in the heart, liver, and kidneys revealed that KD in non-infected mice induced an increase in mitochondrial content. However, this increase was reverted under SARS-CoV-2 infection (Supplementary Fig. 8a–c). RSCs or respirasomes are the active conformation of assembled ETC complex units (CI, CIII, and CIV) that are required for electron transport and respiration. RSC assembly by BN-PAGE is a more accurate indicator of respiratory activity than measuring individual complex subunit levels by Western blot alone^30^. In the C57Bl/6 murine model used in this study, RSCs are comprised by CI + CIII2^31^. We did not observe significant changes in RSC assembly levels in the heart in the KD-CoV-2 condition (Supplementary Fig. 8d, e); however, in the liver, RSC assembly was significantly reduced by almost 50% between the CD-SARS-CoV-2 and KD-SARS-CoV-2 conditions (Fig. 6c).

In order to detect resulting functional differences in fuel capacity we measured the respiratory activity of CI and CII normalized by CIV in frozen tissue homogenates. In accordance with our results probing for protein levels of complexes I and II in the heart, the activity of CII was increased by ~65% in KD-SARS-CoV-2 samples, whereas in the liver we observed a trend towards decreased CI activity under KD-SARS-CoV-2 (62.4% decrease, *p* = 0.15) (Fig. 6d). Curiously, in the heart, the KD alone mildly but significantly caused reduced CI activity, which was not reflected in a change in CII protein levels. In both heart and liver, CI/II activity ratios were normalized to uninfected control animals (Fig. 6e). Interestingly, in the kidney, we observed a significant increase in CI activity by ~170% and a strong trend towards increasing CII activity in KD-SARS-CoV-2 samples (*p* = 0.08) (Supplementary Fig. 8f). Overall, these data indicate restored CI/II ratios in the KD-SARS-CoV-2 group; however, in the kidney, this was driven in the direction of increased CI/CII activity as opposed to the heart and liver (Supplementary Fig. 8g) (uncropped seahorse immunoblots available in Supplementary Fig. 9).

## Discussion

A KD has shown beneficial effects across numerous clinical studies and animal models of disease as a noninvasive means to induce systems-level immune modulation and reduce inflammation^32,33^. In our previous report, we showed that SARS-CoV-2 infection causes multi-organ toxicity, body weight loss, and spleen reduction. Animals showed severely reduced activity, lethargic behavior, and profound morbidity, with mobilization of peripheral fat storages^23^.

Here we show that the implementation of a KD two weeks prior to infection reduces body weight loss, rescues reduction in spleen size and mice activity with overall improved health. These observations are in line with recent reports showing that a KD or the administration of BHB is beneficial to mice survival following SARS-CoV-2 or natural beta coronavirus (mCoV) infection through restored immunity (increased CD4 + T and γδ T cells)^12,34^. Similar findings were reported in the context of influenza infection^12^. The observed increase in spleen weight may be due to increased immune response and accumulation of immune cells in spleen.

The KD causes transcriptional reprogramming at the systems level, with up-regulation of PPARα and its downstream effectors^35^. Our data demonstrate that the implementation of a KD in the uninfected group induces transcriptional changes that resemble those detected following infection in the heart, liver, and kidney. This indicates that a KD shifts the transcriptional baseline toward adaptative changes induced by SARS-CoV-2 infection. Indeed, 327 (heart), 499 (liver), and 341 (kidney) of genes reprogrammed by the KD were consistently changed following SARS-CoV-2 infection in the CD group, indicating that the KD anticipates approximately 35% (heart), 45% (liver) and 32% (kidney) of transcriptional reprogramming observed during infection in CD.

To identify the pathways modulated across KD and SARS-CoV-2 infection, we performed GO enrichment of shared DEGs. This analysis predicted lipid and acetyl-CoA metabolism upregulation in all tissues, with increased transcription of the PPARα gene set. PPARα is the master regulator of lipid metabolism, particularly in the liver. Its activation promotes fatty acid uptake and catabolism through ketogenesis, fatty acid transport, and mitochondrial β-oxidation. PPARα also increases the expression of *CPT1*, which encodes for the carnitine palmitoyltransferase I (CPR1) transporter of fatty acids inside the mitochondria as carnitine conjugates (acyl-carnitines). We observed consistent up-regulation of PPARα and downstream effectors in uninfected animals under KD and in CD-SARS-CoV-2 mice. These data indicate that ketogenesis is activated at the transcriptional level in extrapulmonary tissues following SARS-CoV-2 infection, and are consistent with our previous report showing loss of peripheral adipose tissue, and reduced adipocyte size^23^. Increased acyl-carnitine levels in the liver of infected mice under CD endorse this interpretation. Serum BHB levels were highly heterogeneous across infected mice. Similar results were reported in a recent observational study of COVID-19 patients and in a murine model of SARS-CoV-2^23,36^.

Previous investigations of SARS-CoV-2 reported dysregulation of NAD+ metabolism, increased systemic inflammation, and matrix remodeling following infection^37^. In particular, SARS-CoV-2 causes the dysregulation of NAD+ synthesis and utilization, with increased expression of genes in the NAD+ salvage pathway (*Nmrk1* and *2*), and striking increase of manyl noncanonical mono(ADP-ribosylating) PARPs^27^. Our data show decreased transcription of *Nmrk2* in the heart of animals infected under KD. With *Nmrk1*, *Nmrk2* initiates nicotinamide riboside (NR) conversion to NAD+ *via* NR phosphorylation to nicotinamide mononucleotide (synthesis of NAD+ through salvage metabolism)^38^. In the heart, NAD+ is crucial to mitochondrial homeostasis and oxidative stress and serves as cosubstrate for sirtuin enzymes and poly(ADP-ribose) polymerases^39^. Therefore, in cardiac tissues a KD can mitigate NAD+ salvage induced by the virus^38^. This observation is supported by restored mitochondrial fuel utilization driven by changes in the CI/CII ratio (seahorse analysis), and increased energy currency metabolites (metabolomics analysis).

Our data also show decreased *Timp1*, *Thbs1*, *Tnc*, *Adam8*, *Chil3*, *Mmp-12* and *Mmp-3* in the heart of animals infected under KD. *Timps* encode for a family of glycoprotein inhibitors of *Mmps*, which are released in response to cytokine flow to maintain matrix equilibrium and cytokine shedding during inflammation, and have been reported upregulated in SARS-CoV-2 patients^40^. Tush decreased *Timp1*, *Mmp-12* and *Mmp-3* suggest reduced matrix remodeling and inflammation in the infected KD group. Decreased *Thbs1*, *Tnc*, and *Adam8* (matrix remodeling and cell-matrix interaction), and *Chil3* (stimulation of immune function and inflammation) support this interpretation^41–43^.

We further detected *Saa1*, *Saa2*, *Igfbp1*, *Gdf2*, *Hnf1a*, *Soat2*, *Bco2*, *Amt*, *Acaca* (rate-limiting step in fatty acid synthesis), and *Esr1*, increase in cardiac tissue of KD infected animals, and *Saa1-3* upregulation in the liver. Previous studies reported altered levels of *Saa1* and *Saa2* following SARS-CoV-2 infection^44–46^. *Saa1* and *Saa2* are induced by inflammatory cytokines (IL-1β, IL-6 and TNF-α) in the liver. However, their extrahepatic role is poorly understood. Recent evidence suggests that these proteins may exert immunomodulatory and inflammatory homeostatic functions, and inhibit TNF-α mediated apoptosis^45^.

Taken together, these data indicate that a KD mitigates SARS-CoV-2 induced matrix remodeling and inflammation while reducing NAD+ scavenging.

To validate this interpretation, we measured a robust panel of markers of systemic inflammation in serum. We found that a KD reduces pro-inflammatory cytokines (*i.e*., TNF-α, IL-15, IL-22, G-CSF, M-CSF, MCP-1), metabolic markers of inflammation (i.e., kynurenine/tryptophane ratio), and inflammatory prostaglandins in infected animals. RNA sequencing also showed decreased *IDO2* transcription in the heart. IDO enzymes catalyze the catabolism of tryptophane to kynurenine under inflammatory stimuli, such as IFN-γ signaling. In physiologic conditions, *IDO2* is rarely expressed. However, *IDO2*, rather than the constitutively expressed *IDO1*, has been shown associated with the accumulation of downstream products of kynurenine metabolism and inflammation in the lung, heart, and brain of deceased covid-19 patients^47^.

Previous investigations established increased serum cytokines in COVID-19 patients (i.e., cytokine storm)^48^. Among these, TNF-α, M-CSF, G-CSF have been proposed as predictors of intensive care unit (ICU) requirements and lung injury^49,50^. A further study demonstrated that TNF-α levels in serum can serve as a prognostic marker of post-acute sequelae in COVID-19 patients (long covid)^51^. In addition, the kynurenine/tryptophan ratio positively correlates with patient severity^52,53^. In this context, our data suggest that a KD may improve patient health with reduced risk of hospitalization and long covid^47,54^. On the contrary, a diet rich in carbohydrates may exacerbate the inflammatory response during infection.

Numerous studies corroborate the anti-inflammatory properties of a KD, e.g., in the context of cancer, epilepsy, and neurodegeneration^13,20,55,56^. This has been linked with the inhibition of NLRP3 by BHB in the inflammasome pathway^57–59^. However, we did not detect significant transcriptional modulation of the inflammasome under both CD and KD (Supplementary Fig. 10**)**. This and other discrepancies may be linked to limitations intrinsic to the physiology of the in vivo model used in this study, particularly in relation to differences in hACE2 human expression in infected mice and humans, and to the use of the *i.p*. infection route, which differs from the natural respiratory route in humans.

In our previous work, we observed suppression of OXPHOS and of the TCA at the transcriptional level in infected animals^23^. Independent investigations confirmed similar changes in central carbon metabolism in cohorts of patients and in in vivo*/*in vitro models of infection^60–67^. Our data indicate that a KD increases hepatic metabolic associations with other tissues, a metabolic adaptation also observed in the infected CD group. This suggests that a KD anticipates the systemic metabolic rewiring induced by the virus. Across all tissues, we detected increased energy currency metabolites, lipid precursors, amino acids, and acyl-CoAs. Nucleotide metabolites were also increased in all tissues with a slight change in the heart. Recent literature reported altered amino acid and lipid precursors in plasma from COVID-19 patients, with metabolite levels correlating with disease severity and risk of hospitalization, suggesting that the implementation of a KD may improve patient health^52,53,60,61,68–76^. However, robust follow-up clinical studies are needed to translate these findings into clinical recommendations.

The KD has been shown to rewire mitochondrial metabolism toward ketones and fatty acid oxidation-driven maximal respiratory capacity^29^. We show that SARS-CoV-2 infection in CD mice increases CI/II ratios in the heart and liver. The KD normalizes these changes by regulating CII levels in the heart and respirasome assembly in the liver. In the kidney, SARS-CoV-2 infection decreases CI/II ratios in CD, however; a KD normalizes these ratios. Consistently, we detected rescued energy currency metabolites. Overall, these data suggest that a KD mitigates metabolic dysregulations induced by SARS-CoV-2 infection and confirm the metabolic changes predicted at the transcriptional level.

In summary, our investigation demonstrates that a KD can reprogram and in part anticipate the transcriptional and metabolic changes caused by SARS-CoV-2 infection, with improved mice health, reduced inflammation, and rescued metabolism.

## Methods

### Animal care and use

All animal studies were approved by the Animal Research Committee, University of California, Los Angeles and conducted in compliance with all relevant ethical regulations for animal testing. Male C57Bl/6 mice (000664, Jackson Labs) (14–17 weeks) were housed in a room with controlled temperature and humidity and fed standard chow diets (T.2018.15, Envigo). AAV9-CMV-hACE2 (AAV-200183, Vector Biolabs) viruses were purchased from Vector Biolabs. Animals were injected intravenously with 100 µl injection containing 2 × 10 genomic copies of the virus. This model was previously described^23^.

### Biosafety

All works in this study involving live SARS-CoV-2 virus were approved by the University of California, Los Angeles Institutional Biosafety Committee (IBC). All work with infectious SARS-CoV-2 was conducted in UCLA performance-validated BSL3 facilities, designed adhering to the guidelines recommended by the Biosafety in Microbiological and Biomedical Laboratories (BMBL), the U.S. Department of Health and Human Services, the Los Angeles Department of Public Health (LADPH) and the Centers for Disease Control and Prevention (CDC).

### SARS-CoV2 viruses

SARS-CoV-2 (USA-WA1/2020), was obtained from the Biodefense and Emerging Infections (BEI) Resources of the National Institute of Allergy and Infectious Diseases (NIAID). The virus was passaged in Vero-E6 cells (ATCC) and viral stocks were stored at –80 °C. Virus titer was determined by plaque assay using Vero E6 Cells.

### Keto diet interventions

After 2 weeks of AAV-CMV-hACE2 injection, mice were fed with chow diet (T.2018.15, Envigo) or ketogenic diet (TD.190049, Envigo) ad libitum for 2 weeks^34^. Then animals were housed in BSL-3 facility for the duration of the experiment (*n* = 5/cage) under the same chow/ketogenic diet. 200 μL of SARS-CoV2 (0.5 × 10^6^ PFU/mL) was injected intraperitoneally to each animal. Cage food weight and individual mouse body weight were recorded daily after SARS-CoV-2 virus infection. Animals were euthanized at 7 days SARS-CoV-2 infection.

### Animal movement analysis

Videos of mice infected under chow/ketogenic diets were recorded for 17 s. Mice pixel coordinates were obtained using Adobe After Effects. The tracking software was used to export movement coordinates for each mouse (tracker positioned between mice ears). Next, screen coordinates (CS) were transposed into Cartesian (vertical reflection line = max (Y CS values) - min(Y CS values)/2 + min(Y CS values), Y cartesian values = −1*(Y CS values - Vertical reflection line) + Vertical reflection line). Keto mice x (length) cartesian values and the modified keto mice Y (width) were set to start at zero. Data were plotted in R Studio using the ggplot2 package, distances traveled between video frames were computed using the dist() function in R.

### Histology

Tissues were fixed in 4% paraformaldehyde and subsequently subjected to paraffin section preparation. Paraffin-embedded tissues were sectioned at 5μm thickness and stained with hematoxylin and eosin (HE staining). Images were taken using Nikon Eclipse Ti2 microscopy (Nikon,USA) with DS-Ri2 brightfield camera.

### Tissue and serum metabolomics

Sample preparation: for metabolomic analysis of the heart, kidney, and liver of chow and keto diet animals: 10 mg tissue was homogenized in 1 ml 80% methanol and incubated for 30 min on dry ice. homogenate was further vortexed and centrifuged at 15,000 × *g* for 15 min at 4 °C. The supernatant was subsequently vacuum-dried for LC/MS.

For metabolic profiling of serum, 50 μL of serum was mixed with 50 μL H_2_O and 400 μL methanol, the sample vortexed and incubated at −80 °C for 20 min. Samples were centrifuged at 15,000 × *g* for 15 min, 4 °C. The supernatant was mixed with 300 µl H_2_O and 400 µl chloroform and thoroughly homogenized for 1 min. The aqueous phase was harvested and dried by a vacuum evaporator.

LC-MS analysis: Dried metabolites were resuspended in 50 μl 50% ACN:water. For HILIC analysis, 5 ml was loaded onto a Luna NH2 3 μm 100 A (150 × 2.0 mm) column (Phenomenex) using a Vanquish Flex UPLC (Thermo Scientific). The chromatographic separation was performed with mobile phases A (5 mM NH4AcO pH 9.9) and B (ACN) at a flow rate of 200 μl/min. A linear gradient from 15% A to 95% A over 18 min was followed by 7 min isocratic flow at 95% A and re-equilibration to 15% A. Metabolites were detected with a Thermo Scientific Q Exactive mass spectrometer run with polarity switching (+3.5 kV/− 3.5 kV) in full scan mode using a range of 70-975 m/z and 70.000 resolution. Maven (v 8.1.27.11) was used to quantify the targeted polar metabolites by AreaTop, using expected retention time and accurate mass measurements (<5 ppm) for identification.

Data analysis, including principal component analysis and univariate *t* test (two-tailed, unequal variance), correlation analysis, heatmap generation, and circos plot visualization was performed by R Studio and in-house scripts^77^.

### Lipidomics

Polar lipids were measured by reversed-phase liquid chromatography (RP-LC) coupled with untargeted high-resolution mass spectrometric detection, with a Thermo Scientific Q Exactive mass spectrometer run in polarity switching (+3.5 kV/− 3.5 kV) in full scan mode. LC separation was performed on a Phenomenex Kinetex C18 1.8 mm (100 × 2.1 mm) UPLC column (Part number 00D-4475-AN) with a Waters UPLC BEH guard column at 50 Celsius and 0.150 mL/min flow rate, 18 min total run time. Mobile phase gradient was as follows: (A) 0.1% formic acid in water; (B) 0.1% formic acid in acetonitrile, starting from 1% B (0–1 min), 99% (10–15 min), 1%B (17–18 min). Raw data were analyzed in XCMS. Significant metabolic features were filtered by t-test significance threshold (adjusted for multiple testing error) *p* < 0.01 significance, retention time >2 min, max intensity >5,000,000 counts (ESI+) or >1,000,000 (ESI-), and fold change >2. Lipids were annotated at the class level by accurate match (5 ppm error) with METLIN, HMDB^78,79^, and by analysis of MS/MD fragmentation spectra.

### Luminex assay for cytokines

Serum samples were harvested on day 7 from infection and stored at −80 °C until analysis. Pro-inflammatory cytokines were measured using the technologyCytokine & Chemokine 36-Plex Mouse ProcartaPlex™ Panel 1 A (Invitrogen) that enables the analysis of 36 mouse pro-inflammatory cytokines and chemokines in a single well by Luminex xMAP.

Luminex xMAP technology was used for readout acquisition as described in previous work^80^. Statistical analysis was performed by univariate t-testing (two-tailed, heteroschedastic distribution) with significance threshold *p* < 0.05.

### Tissue seahorse analysis

Respirometry assay using RIFS: tissue homogenates were loaded into Seahorse XF96 microplates in 20 µL of MAS and centrifuged at 1500 x g for 5 min at 4 °C. Subsequently, an additional 130 µL of MAS containing cytochrome c (100 µg/ml) were added to each well and respirometry was assessed using a Seahorse XF96 analyzer (Agilent). The following substrates were injected: Port A: NADH (1 mM) or 5 mM succinate + rotenone (5 mM + 2 μM); Port B: rotenone + antimycin A (2 μM + 4 μM); Port C: N,N,N’,N’-Tetramethyl-p-phenylenediamine (TMPD) + ascorbic acid (0.5 mM + 1 mM); Port D: sodium azide (50 mM). The following amounts of tissue lysates were loaded into individual wells: Liver 4 µg, heart 2 µg, kidney 4 µg. Mitochondrial content was assessed using Mitotracker™ Deep Red FM (MTDR, Thermo Fisher Scientific) was assessed in a sister plate as previously described.

Respirometry data analysis: OCR rates were normalized by protein amounts or MTDR signal using Wave software (Agilent) and traces were exported to GraphPad Prism 9.4.1. Complex I, II, and IV dependent respiration was determined after subtraction of substrate induced respiration by antimycin A (CI, CII) or sodium azide baselines (CIV) respectively. Normalized respiration values were used to calculate rates of fuel preference.

Protein gel electrophoresis and immunoblotting - *SDS-PAGE -* 5-20 µg of total tissue lysates were loaded onto 10-well 4%-12% Bis-Tris precast gels (ThermoFisher Scientific) and gel electrophoresis was performed in xCell SureLock chambers (Novex) under constant voltage at 120 V for 80-90 min.

Blue Native gel electrophoresis: tissue lysates (100-150 µg) were centrifuged at 10,000 × *g* for 10 min. Pellet containing mitochondria was resuspended in 30–50 µl of MAS and protein amount was determined by BCA. Digitonin (DIG) incubation (Liver and Kidney, 3 mg DIG/mg protein; Heart 6 mg DIG/mg protein) was performed on ice for 5 min and then centrifuged at 20,000 × *g* for 30 min as previously described^81,82^. 3 µL of 2.5% Coomassie G-250 was added to the resulting supernatant, samples were loaded onto 3-12% NativePAGE Bis-Tris precast gels (ThermoFisher Scientific), and electrophoresis was performed at 4 °C in xCell SureLock chambers under constant voltage of 20 V for 60 min followed by 200 V for 120 min.

Immunoblotting: proteins were transferred to a methanol-activated PVDF membrane in an xCell SureLock chamber under 100 V constant voltage for 75 min at 4 °C. For native blots, Coomassie staining was completely removed from the membrane using 100% methanol. All washes were done using PBST (1 mL/L Tween-20/PBS). Blots were blocked using 5% BSA in PBST for 1 h and incubated with primary antibodies diluted in blocking buffer overnight at 4 °C. The next day, blots were washed 3 × 5 min and incubated with secondary antibodies conjugated to HRP in blocking buffer for 1 h at room temperature. Subsequently, membranes were washed 3 × 5 min and developed using enhanced chemoluminescence solution (Millipore, WBKLS0500). ECL signal was detected using a ChemiDoc Molecular Imager (BioRad). Band densitometry was quantified using ImageJ Gel Plugin (NIH). We used the following antibodies: Vinculin, from Sigma Aldrich (V9131), mtOXPHOS cocktail (for combined Atp5b, Uqcrc2, Mtco1 and Sdhb detection; ab110413) from Abcam, Ndufb8 (459210) from Thermo-Fisher Biotechnology; Uqcrc2 (14742-1-AP) from Proteintech and Tomm20 (sc-11415) from Santa Cruz Biotechnology.

### RNA sequencing

Total RNA was extracted using RNeasy Plus Mini Kit (74134, Qiagen). Libraries were prepared by the Technology Center for Genomics & Bioinformatics at UCLA using Illumina TruSeq Stranded Total RNA Sample Prep kit and sequenced with 50 bp single end reads on an Novaseq S4.

### Analysis of RNA sequencing data

The reads were mapped with STAR 2.5.3a to the human genome (hg38) for the cultured human cell libraries or mouse genome (mm10) for the mouse cell libraries. The counts for each gene were obtained using quantMode GeneCounts in STAR commands, and the other parameters during alignment were set to default. Differential expression analyses were carried out using DESeq2 with default parameters. Counts normalized by sequencing depth were obtained using DESeq2 estimate size Factors function with default parameters. Genes with adjusted *p* value < 0.05 and FC > ±1 were considered significantly differentially expressed. Significantly up-regulated or down-regulated genes were uploaded to the Enrichr platform for analyses. Transcription factor regulatory network analysis of RNA-seq data was performed by the transcriptional factor (TF)-target interaction database Transcriptional Regulatory Relationships Unraveled by Sentence-based Text mining (TRRUST, v2)^25^. Enrichment analysis for DEGs was performed using the DESeq2 method with FDR cutoff 0.1 and minimum fold change 2, and gene sets from the GO Biological Process repository. Two nodes (pathways) are connected if they share >30% genes. Further RNA-seq analysis (e.g., principal component analysis) was performed in iDEP.95^83^.

### Statistics and reproducibility

All data are presented as mean ± standard error (S.E.M.) and mentioned in the figure legends. Statistical analysis was performed using GraphPad (Prism) software using Student’s *t* test (two tailed) or ordinary one-way ANOVA with Tukey’s multiple comparison test. A *p* value < 0.05 was considered statistically significant. All data were acquired with a minimum of three biological replicates. Reproducibility of methos was tested by a minimum of three technical replicates of standard measurements.

### Reporting summary

Further information on research design is available in the Nature Portfolio Reporting Summary linked to this article.

### Supplementary information

Supplementary Information

Description of Additional Supplementary Files

Supplementary Data 1

Supplementary Data 2

Supplementary Data 3

Supplementary Data 4

Supplementary Data 5

Supplementary Data 6

Supplementary Data 7

Supplementary Data 8

Supplementary Data 9

Supplementary Video 1

Supplementary Video 2

Reporting Summary

## Data Availability

RNA-sequencing data are available in GSE243426. All other data are available in Supplementary Data 9 and from the corresponding authors on reasonable request. Unedited blots/gel images are available in the Supplementary Information.
