# Supplementary material for: A ketogenic diet can mitigate SARS-CoV-2 induced systemic reprogramming and inflammation
Source: Commun Biol. 2023 Nov 3;6:1115. doi: 10.1038/s42003-023-05478-7 (PMC10624922; doi:10.1038/s42003-023-05478-7)
Supplement: Supplementary file 1 — Supplementary Information [file 42003_2023_5478_MOESM1_ESM.pdf]

Supplementary Information
to

A ketogenic diet can mitigate SARS-CoV-2 induced systemic reprogramming and inflammation

Amelia Palermo'[1,2,3,4], Shen Li'[5,6,7,8,9,10], Johanna ten Hoeve[2,3,4], Akshay Chellappa[1], Alexandra Morris[1], Barbara Dillon[11], Feiyang Ma[12], Yijie Wang[6,7,8,9,10], Edward Cao[6,7,8,9,10], Byourak Shabane[13], Rebeca Acín-Perez[13], Anton Petcherski[13], A. Jake Lusis[1,5], Stanley Hazen[14], Orian S Shirihai[1,13], Matteo Pellegrini[7,8,9], Vaithilingaraja Arumugaswami[1,8], Thomas G. Graeber[1,2,3,4,8]*, Arjun Deb[2,5,6,7,8,9]*

*Corresponding authors
'These authors contributed equally
[1]Department of Molecular and Medical Pharmacology, David Geffen School of Medicine, University of California, Los Angeles CA 90095, [2]California Nanosystems Institute, University of California, Los Angeles, CA 90095, [3]UCLA Metabolomics Center, University of California, Los Angeles, [4]Crump Institute for Molecular Imaging, University of California, Los Angeles, [5]Division of Cardiology, Department of Medicine, David Geffen School of Medicine, University of California, Los Angeles, CA 90095., [6]UCLA Cardiovascular Research Theme, David Geffen School of Medicine, University of California, Los Angeles, CA 90095, [7]Department of Molecular, Cell and Developmental Biology, Division of Life Sciences, University of California, Los Angeles, CA 90095, [8]Eli & Edythe Broad Center of Regenerative Medicine and Stem Cell Research, University of California, Los Angeles, CA 90095, [9]Molecular Biology Institute, University of California, Los Angeles, CA 90095, [10]Department of Genetics, David Geffen School of Medicine, Los Angeles, CA 90095, [11]Department of Environment, Health and Safety, University of California, Los Angeles, CA 90095, [12]Division of Rheumatology, Department of Internal Medicine, University of Michigan, Ann Arbor, Michigan, USA 48109, [13]Department of Medicine, Endocrinology, David Geffen School of Medicine, University of California, Los Angeles, CA 90095, USA, [14]Department of Cardiovascular and Metabolic Sciences, Cleveland Clinic, Cleveland, OH 44195.

INDEX

Supplementary Figure 1. Schematization of ketogenic metabolism**.**

Supplementary Figure 2. Glucose, β-hydroxybutyrate (BHB) and body weight in KD for WT, hACE2+ mice.

Supplementary Figure 3. Principal component analysis, volcano plot and GO enrichment analysis of RNA-sequencing data (liver) in hACE2+ KD vs hACE2+ CD uninfected mice.

Supplementary Figure 4. Glucose and BHB in serum in hACE2+ CD or hACE2+ KD infected mice.

Supplementary Figure 5. PPARα gene set enrichment, acylcarnitine (liver),TRRUST analysis, serum BHB at day 7 from infection in hACE2+ CD mice.

Supplementary Figure 6. GO enrichment analysis of RNA-sequencing data (heart) in hACE2+ KD *vs* hACE2+ infected mice.

Supplementary Figure 7. Principal component analysis of metabolomics (heart, serum, kidney, liver) in hACE2+ CoV-2 infected and control mice in KD or CD.

Supplementary Figure 8. Seahorse analysis supplementary data.

Supplementary Figure 9. Uncropped seahorse immunoblots.

Supplementary Figure 10. NLRP3 inflammasome transcriptional gene set analysis for infected mice under CD or KD.

Ketogenesis

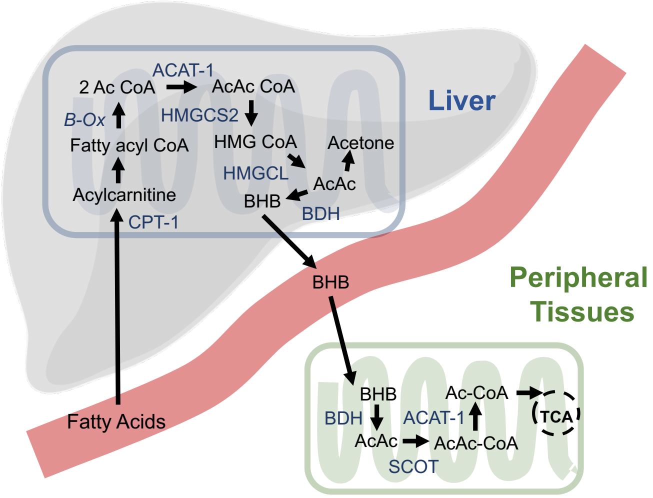

Ketolysis

Supplementary Figure 1. Schematization of ketogenic metabolism.

a

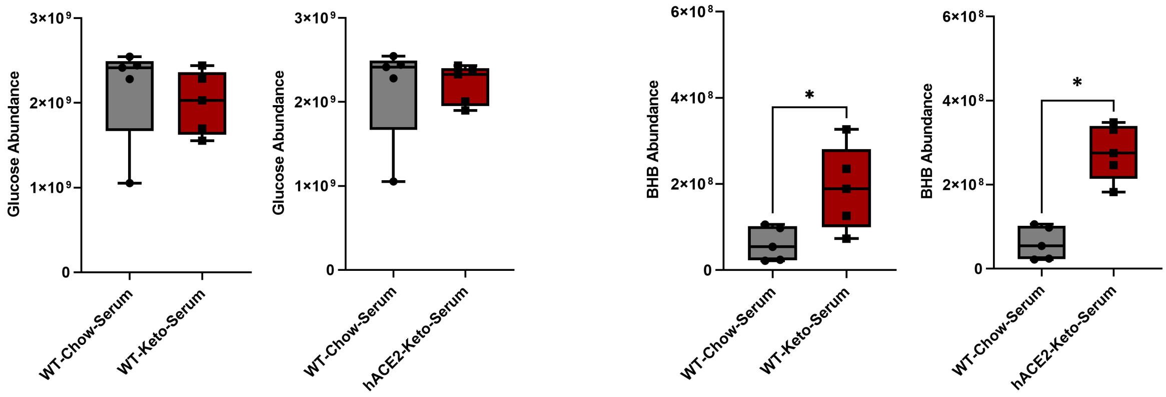

b

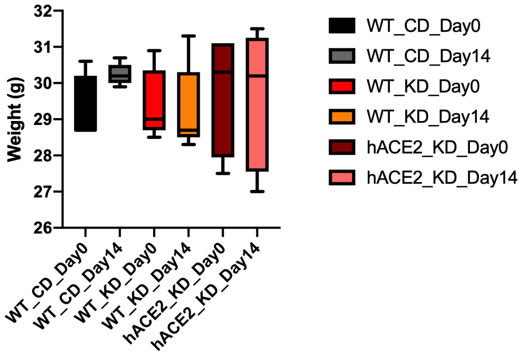

Supplementary Figure 2. a) glucose and β-hydroxybutyrate (BHB) abundance in CD, KD WT and hACE2+ KD mice in serum (* for significance $p < 0.05$, *n*=5/group); **b)** Body weight changes during KD induction in WT and hACE2+ mice, and CD control (followed over 2 weeks of diet).

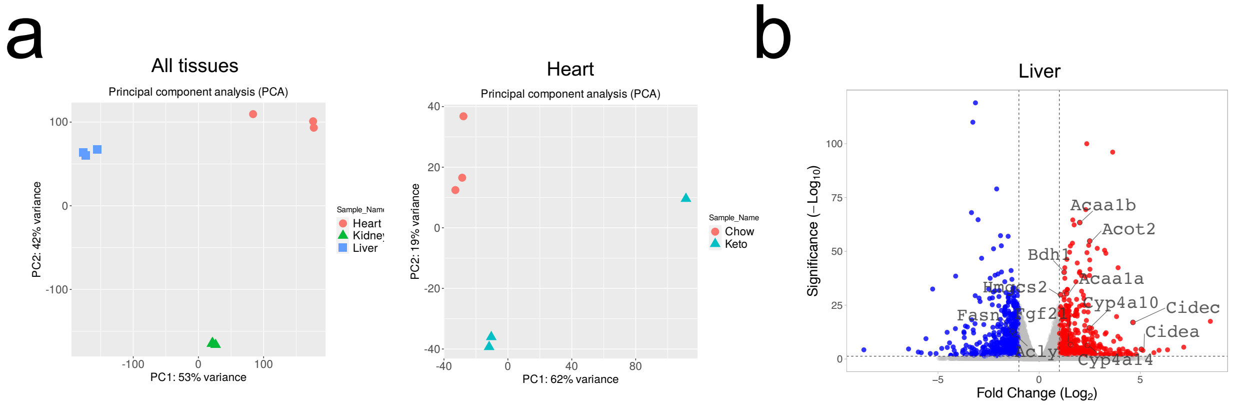

Gene	Log2FC	FC	Adjp
**Ketogenesis**			
Acat1	0.86	1.82	4.27E-21
Bdh1	1.23	2.35	4.71E-41
Ucp2	0.95	1.93	1.99E-14
Hmgcl	0.93	1.91	5.19E-18
Hmgcs2	1.05	2.07	1.19E-30
Cpt1a	0.83	1.78	7.14E-22
Cpt2	0.74	1.67	3.70E-13
Ppara	0.15	1.11	0.43
Fgf21	1.55	2.94	2.05E-07
**Lipid β-ox /PPARα targets**			
Acot2	2.50	5.66	1.46E-55
Cidea	5.05	33.19	3.02E-05
Cidec	4.64	24.90	1.09E-17
Cyp4a10	2.48	5.59	2.89E-15
Cyp4a14	2.39	5.23	4.71E-07
Acox1	0.95	1.94	1.77E-23
Hsd17b4	0.52	1.43	8.17E-10
Acaa1a	1.36	2.56	3.81E-31
Acaa1b	2.01	4.02	4.30E-64
**Lipid synthesis**			
Fasn	-1.30	0.41	1.46E-13
Scd1	-4.14	0.06	7.45E-15
Srebf1	-0.10	0.93	0.64
Acly	-1.21	0.43	4.10E-12

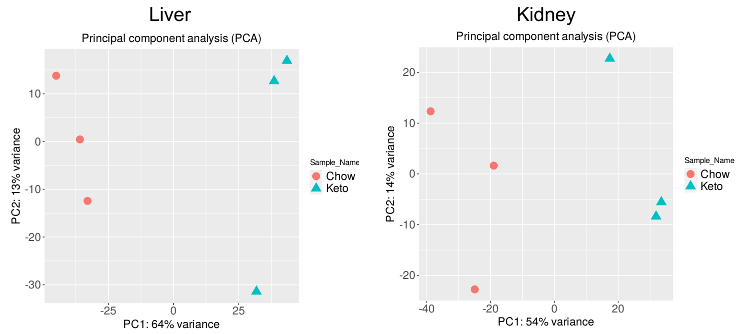

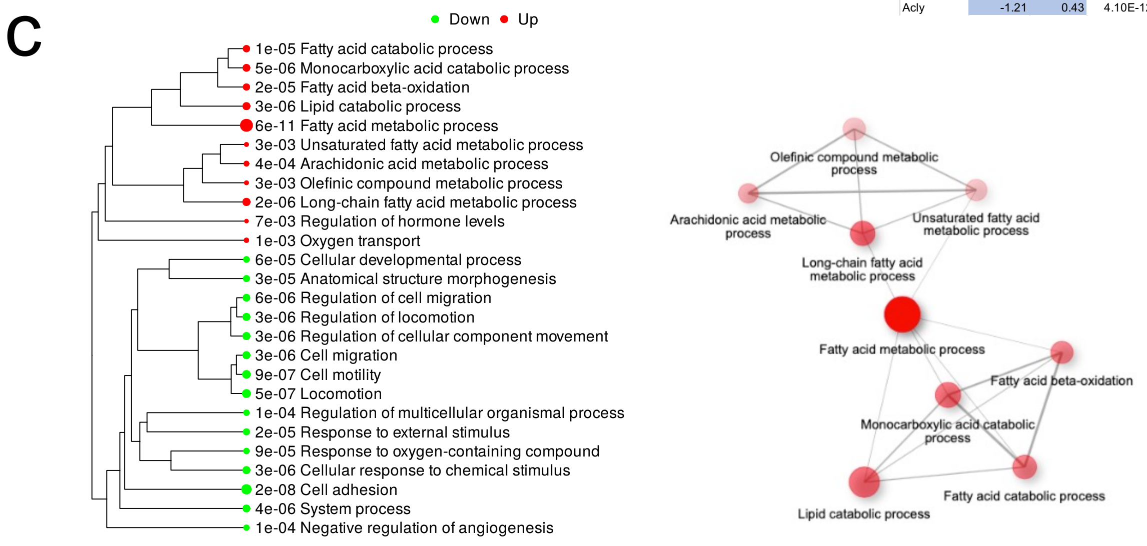

Supplementary Figure 3. a) principal component analysis of RNA-seq data for heart, liver and kidney in hACE2+ mice under CD or KD (both uninfected); **b)** volcano plot of differentially regulated genes in hACE2+ KD *vs* hACE2+ CD mice (both uninfected) and table showing log2FC

and adjusted p values for up- and down-regulated genes in the ketogenesis, lipid β-oxidation/PPAR-α targets and lipid synthesis pathways; **c)** GO gene set network enrichment analysis of differentially regulated genes in RNA-sequencing data (liver) in hACE2+ KD Mock *vs* hACE2+ CD Mock mice (red, upregulated pathways, green, downregulated pathways).

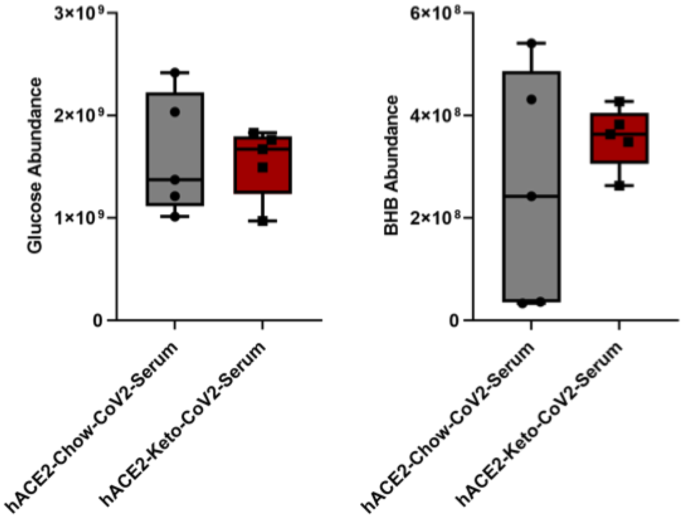

Supplementary Figure 4. Glucose and β-hydroxybutyrate (BHB) levels in mice serum in CD or KD SARS-CoV2 infected mice (*n*=5/group, significance threshold $p < 0.05$).

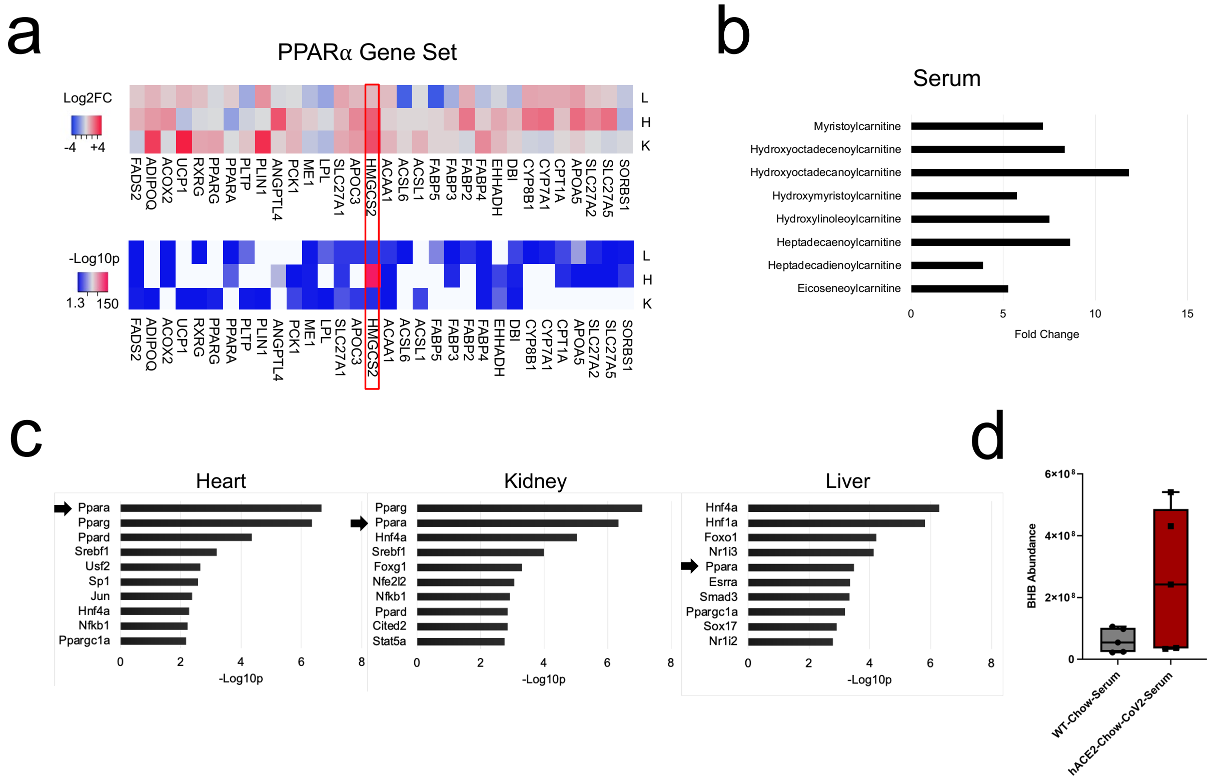

Supplementary Figure 5. a) PPARα gene set enrichment analysis for liver, heart and kidney tissues at 7 days from infection showing significant up regulation of hydroxymethylglutaryl-CoA synthase 2 (HMGCS2) across all tissues in CD mice (n=3/group, $p < 0.05$ and fold change > |1|); **b)** fold change of acylcarnitine abundances in mice liver after 7 days from infection in CD (significance p <0.05, fold change > |2|) (n=5/group); **c)** transcriptional regulatory network analysis of RNA-sequencing data (first 500 DEGs ranked according to adjusted significance p value) from mice liver, heart, and kidney, after 7 days from infection with SARS-CoV2, constructed using the TRRUST v2 dataset. Bars represent -log10 of transcriptional regulatory network significance, arrows highlight the PPARα transcriptional regulatory network for each tissue (n=3/group); **d)** serum levels of 3β–hydroxybutyrate (BHB) after 7 days from infection in CD mice (n=5/group).

Hearth KD CoV-2 vs CD CoV-2				
Direction	adj.Pval	nGenes	Pathways	Genes
**Down regulated**	**0.00116021**	**11**	**Defense response**	**Timp1, Hmox1, Adam8, Lcn2, Lrp8, Clec4d , Ccl2, Thbs1, Chil3, Mmp12, Lgals3,**
**Down regulated**	**0.00116021**	**3**	**Eosinophil migration**	**Adam8, Ccl2, Lgals3**
**Down regulated**	**0.00217031**	**10**	**Regulation of immune system process**	**Hmox1, Fstl3, Adam8, Lrp8, Gpnmb, Clec4d, Ccl2, Thbs1, Mmp12, Lgals3**
**Down regulated**	**0.00217031**	**7**	**Regulation of leukocyte activation**	**Hmox1, Adam8, Gpnmb, Clec4d, Ccl2, Thbs1, Lgals3**
**Down regulated**	**0.00312756**	**6**	**Wound healing**	**Timp1, Hmox1, Tnc, Ccl2, Thbs1, Mmp12**
Down regulated	0.00453005	3	Regulation of extrinsic apoptotic signaling pathway via death domain receptors	Hmox1 Thbs1 Lgals3
**Down regulated**	**0.00453005**	**4**	**Regulation of tumor necrosis factor superfamily cytokine production**	**Adam8 Gpnmb Ccl2 Thbs1**
Down regulated	0.00453005	2	Regulation of blood vessel endothelial cell proliferation involved in sprouting angiogenesis	Hmox1 Thbs1
Down regulated	0.00453005	8	Regulation of cell motility	Timp1 Hmox1 Adam8 Gpnmb Ccl2 Thbs1 Mmp12 Lgals3
Down regulated	0.00453005	5	Negative regulation of cell adhesion	Adam8 Tnc Gpnmb Thbs1 Mmp12
Down regulated	0.00453005	9	Response to biotic stimulus	Ankrd1 Adam8 Lcn2 Lrp8 Clec4d Mt2 Ccl2 Mmp12 Lgals3
Down regulated	0.00453005	8	Cell activation	Timp1 Hmox1 Adam8 Gpnmb Clec4d Ccl2 Thbs1 Lgals3
Down regulated	0.00453005	7	Regulation of cell adhesion	Fstl3 Adam8 Tnc Gpnmb Ccl2 Thbs1 Mmp12
Down regulated	0.00453005	2	Wound healing involved in inflammatory response	Timp1 Hmox1
Down regulated	0.00453005	2	Eosinophil chemotaxis	Ccl2 Lgals3
**Up regulated**	**1.80E-06**	**11**	**Steroid metabolic process**	**Cyp17a1, Esr1, Srd5a1, Soat2, Tm7sf2, Hmgcs2, Hnf1a, Scd1, Serpina12, Dhcr7, Saa1**
**Up regulated**	**9.11E-05**	**7**	**Cholesterol metabolic process**	**Soat2 Tm7sf2 Hmgcs2 Hnf1a Serpina12 Dhcr7 Saa1**
**Up regulated**	**0.00011135**	**20**	**Small molecule metabolic process**	**Cyp17a1 Acaca Srd5a1 Soat2 Acat2 Tm7sf2 Dhtkd1 Hmgcs2 Hnf1a Bco2 Amt Scd1 Serpina12 Nags Dhcr7 Cyp2c70 Gdf2 Cyp4f15 Saa1 Urad**
**Up regulated**	**0.00071679**	**6**	**Steroid biosynthetic process**	**Cyp17a1 Srd5a1 Tm7sf2 Hmgcs2 Hnf1a Dhcr7**
**Up regulated**	**0.00189709**	**13**	**Cellular lipid metabolic process**	**Esr1 Acaca Srd5a1 Soat2 Acat2 Hmgcs2 Hnf1a Pik3c2g Bco2 Scd1 Serpina12 Cyp2c70 Cyp4f15**
Up regulated	0.00307265	9	Organic hydroxy compound metabolic process	Srd5a1 Soat2 Tm7sf2 Hmgcs2 Hnf1a Serpina12 Dhcr7 Cyp4f15 Saa1
Up regulated	0.00421454	5	Olefinic compound metabolic process	Cyp17a1 Srd5a1 Bco2 Cyp2c70 Cyp4f15

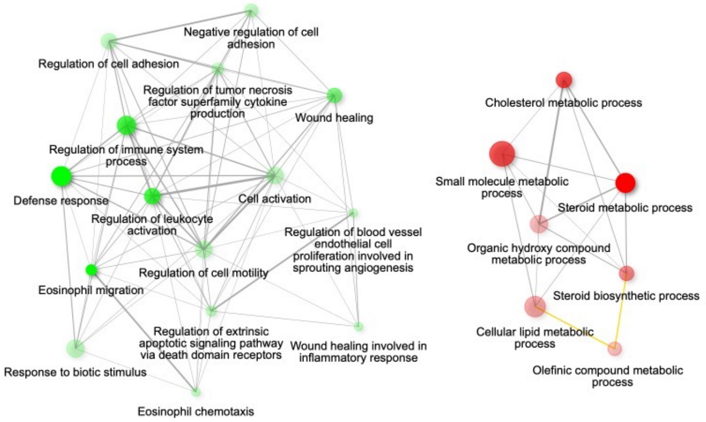

Supplementary Figure 6. GO gene set network enrichment analysis of differentially regulated genes in RNA-sequencing data in heart in SARS-CoV2 infected mice (KD *vs* CD). Enrichment analysis for DEGs was performed using the DESeq2 method with FDR cutoff 0.1 and minimum fold change 2, and gene sets from the GO Biological Process repository. Two nodes (pathways) are connected if they share >30% genes, red represents up regulated pathways, darker nodes are more significantly enriched gene sets, bigger nodes represent larger gene sets, ticker edges represent more overlapping genes.

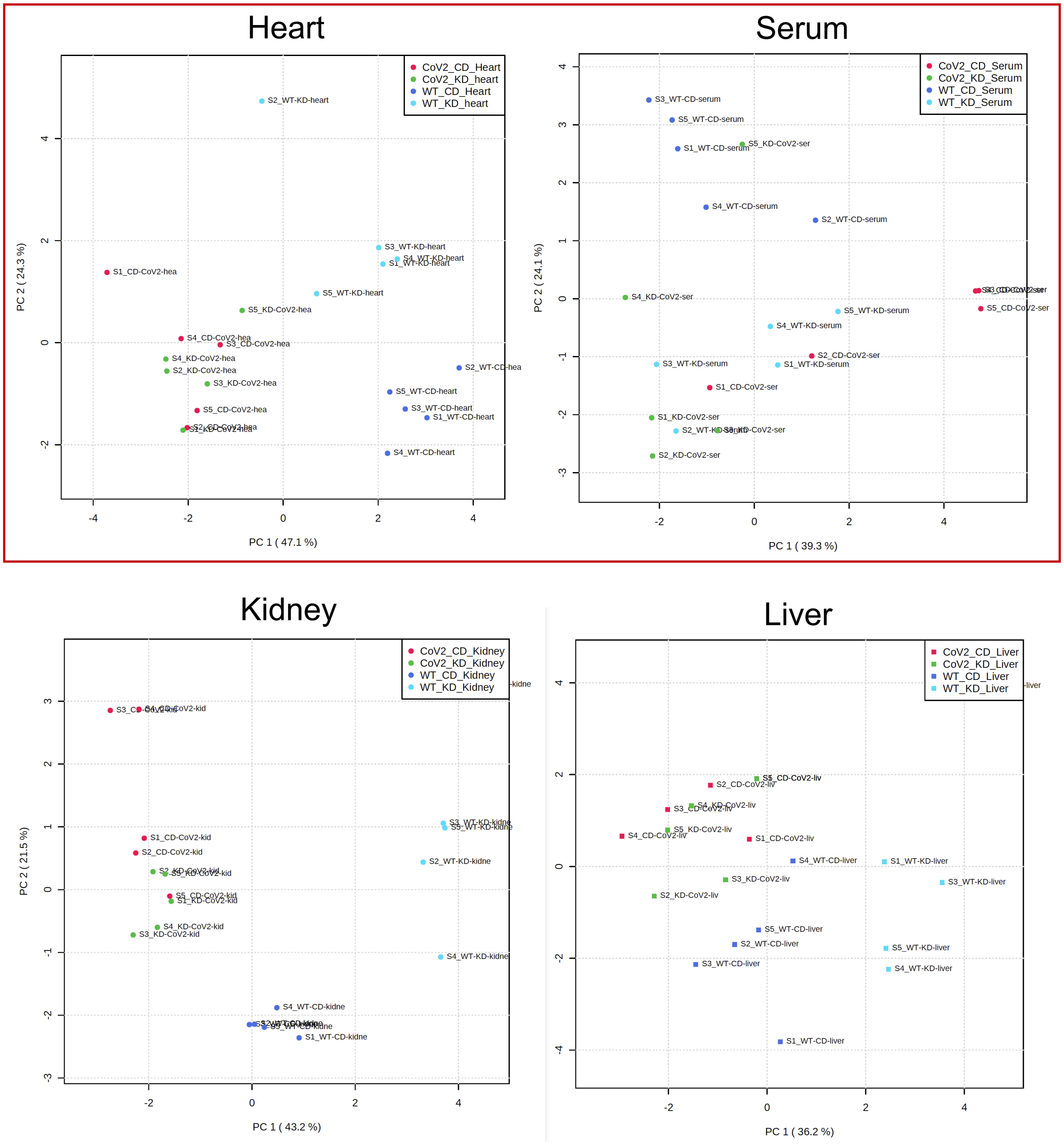

Supplementary Figure 7. Principal component analysis of metabolomics data showing significant changes in at least one tissue/condition ($p < 0.05$ and FC > |1|) for heart, serum, kidney, and liver in SARS-CoV-2 infected/uninfected (control) mice under KD or CD (*n*=5/group).

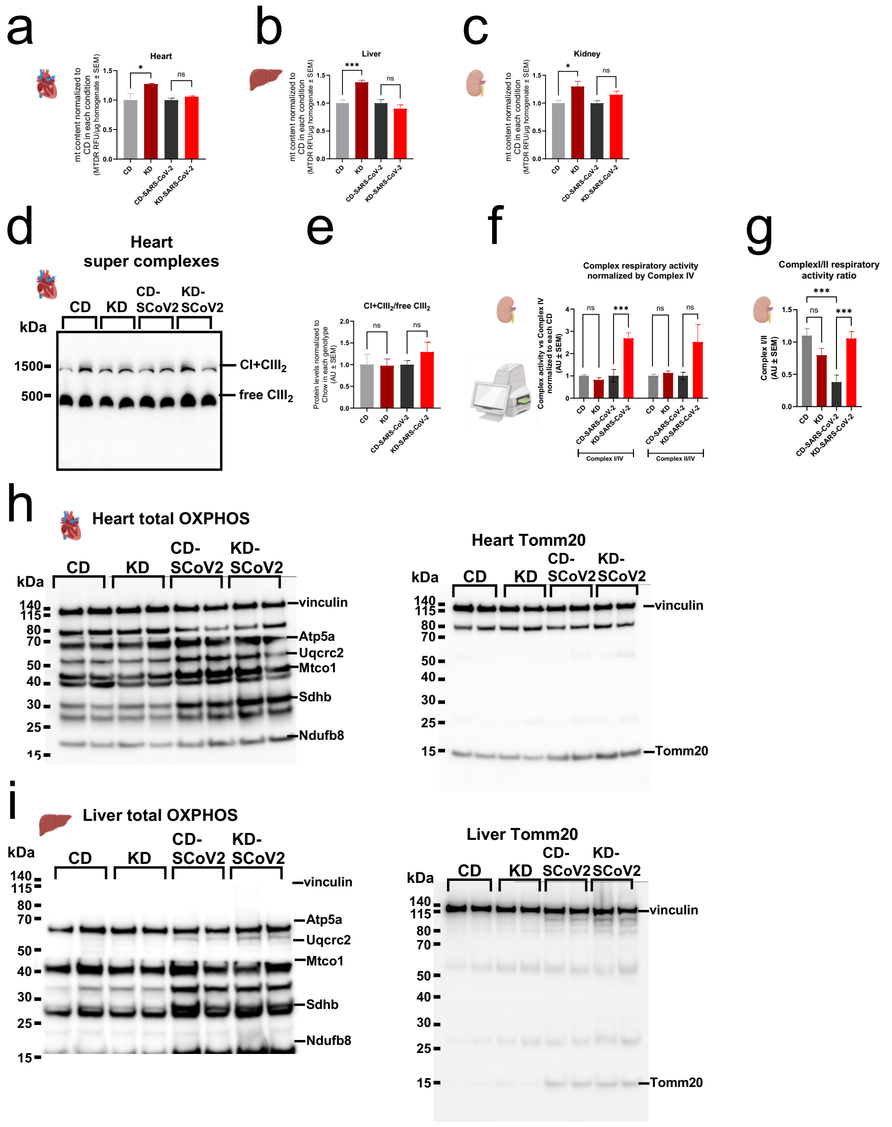

Supplementary Figure 8. Mitochondrial mass analysis in **a)** heart, **b)** liver, **c)** kidney. **d)** Immunoblot of blue native gel electrophoresis stained for Uqcrc2 in the heart. **e)** Quantification of assembled CI+CIII2 versus unassembled CIII2 respirasome ratios in the heart normalized to each CD condition. Graphs represent mean ±SEM of N=4 biological replicates. **f)** Respiratory activity of complexes I and II normalized by complex IV and each CD condition in the kidney measured by RIFS. Graphs represent mean ±SEM of N=5-10 biological replicates. **g)** Respiratory ratios of complex I/II in the kidney. Graphs represent mean ±SEM of N=5-10 biological replicates. **h, i)** Whole membrane images of total OXPHOS staining (complex I=Ndufb8, complex II=Sdhb, complex III=Uqcrc2, complex IV=Mtco1, complex V=Atp5a, Tomm20) and vinculin from heart and liver. Statistical significance for pairwise comparisons in A, B, C, E, and F was determined using unpaired 2-tailed Student's t-test or Mann-Whitney test where appropriate. Statistical significance of group comparisons in G was determined by One-way ANOVA with Holm-Šídák's multiple comparisons test. *=p<0.05, **=p<0.01, ***=p<0.001.

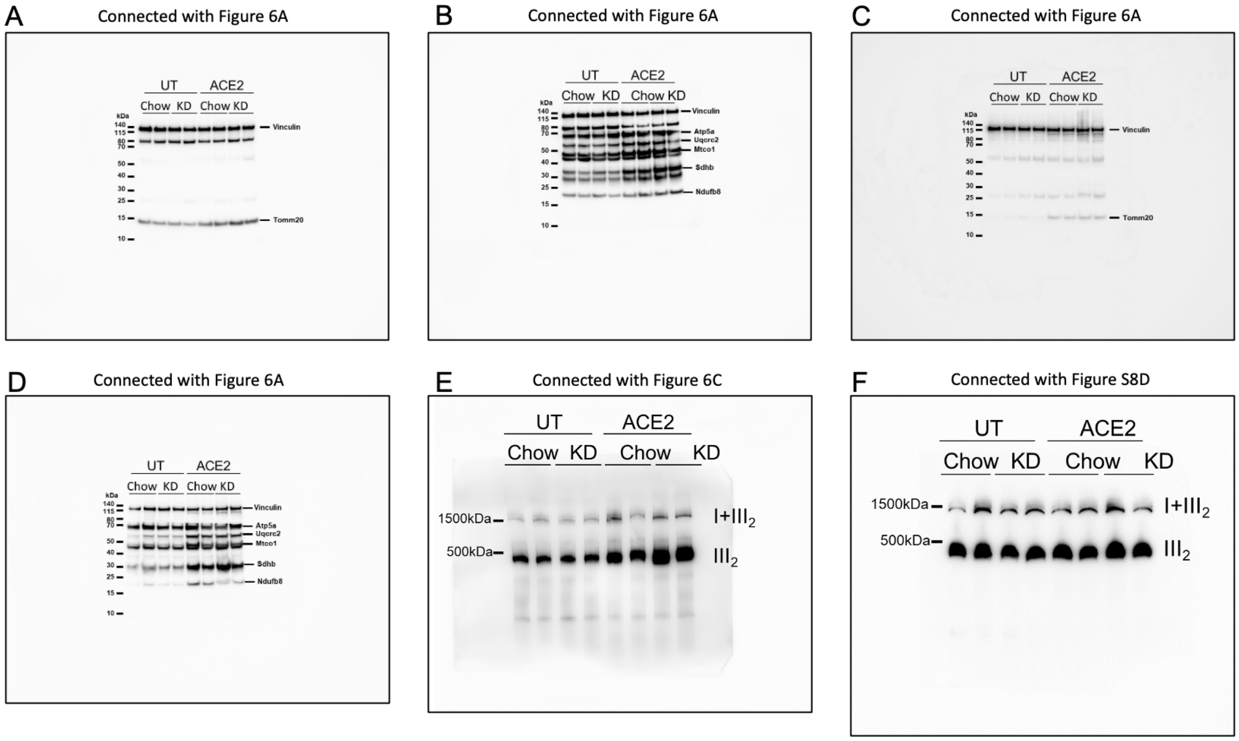

Supplementary Figure 9. a-f) uncropped seahorse immunoblots.

(CoV+ vs Mock) CD	Liver		Kidney		Heart	
Gene	log2FoldCha	padj	log2FoldCha	padj	log2FoldCha	padj
App	-0.3423153	0.00483703	-0.238524	0.0054805	-0.0446387	0.7856388
Casp1	-0.1093449	0.7651363	0.818861	0.03854891	0.3002827	0.5885465
Hsp90ab1	0.3233307	0.03626698	-0.5408839	3.5627E-07	-0.195772	0.09447505
Mefv	-0.1652302	0.8009388	0.1839004	0.9303023	-1.627225	0.3714338
Nlrp3	0.5238939	0.2897234	0.3420884	0.6782744	0.1504231	0.8987734
P2rx7	-0.3889939	0.1690553	0.2183965	0.5485049	-0.1858189	0.5549637
Panx1	-0.1365616	0.890656	0.3118183	0.6757288	0.01509982	0.9867074
Pstpip1	0.5233991	0.6164818	-0.0121071	0.9899481	0.07730529	0.9294771
Pycard	-0.2876032	0.6650046	0.08115971	0.8126235	-0.8352595	0.0025423
Sugt1	-0.0480264	0.8199031	-0.14305	0.2834837	0.1887355	0.274736
Txn1	0.1468964	0.3849401	-0.011905	0.9402218	0.3965358	0.01726207
Txnip	-0.7109239	0.00205565	0.94375542	5.1151E-17	1.358478	0.00560716
(CoV+ vs Mock) KD	Liver		Kidney		Heart	
Gene	log2FoldCha	padj	log2FoldCha	padj	log2FoldCha	padj
App	-0.0900725	0.6216856	-0.2873438	0.00158866	-0.1307284	0.6251281
Casp1	0.95826911	0.00050974	1.3639762	0.00229168	0.2272381	0.7156191
Hsp90ab1	0.1536993	0.2454463	-0.4073162	9.59E-06	-0.0437195	0.851374
Mefv	0.6655186	0.5156681	-0.0239098	0.5	-0.1954177	0.5
Nlrp3	1.49572097	9.37E-05	0.3343609	0.7462106	0.6352095	0.6748814
P2rx7	-0.0481552	0.9063406	-0.1650734	0.6885929	-0.1651845	0.790371
Panx1	0.6544571	0.3913517	0.7336121	0.2883224	0.07396773	0.9536502
Pstpip1	0.7776461	0.3616439	-0.0733855	0.9357134	0.3401629	0.7749411
Pycard	0.3681263	0.5261097	0.60198056	0.00701464	-0.4982977	0.3441938
Sugt1	0.0060055	0.9828179	0.1892025	0.1758379	-0.0574606	0.8659103
Txn1	-0.2943571	0.03422323	0.01261635	0.9539017	-0.0863247	0.8135332
Txnip	-0.3579914	0.3803984	0.1414272	0.3366593	0.1910114	0.3029226
(CoV+ KD vs Mock CD)	Liver		Kidney		Heart	
Gene	log2FoldCha	padj	log2FoldCha	padj	log2FoldCha	padj
App	-0.0458794	0.9442773	0.00017191	0.9997431	0.00694549	0.9974641
Casp1	0.83098482	0.0273742	0.4409857	0.7344761	0.06868844	0.9783912
Hsp90ab1	-0.0203087	0.9859957	0.24088217	0.30376346	0.08495171	0.8707698
Mefv	-0.6621344	0.5	-0.5956393	0.5	-0.1259762	0.5
Nlrp3	0.3953297	0.7403654	0.06067101	0.5	0.15296	0.9728533
P2rx7	0.2412895	0.8244944	-0.1564205	0.9482943	-0.0603667	0.9783419
Panx1	0.6120604	0.5	0.3964755	0.5	0.01937028	0.9974641
Pstpip1	0.7151182	0.5	0.02577697	0.9972775	-0.3726863	0.8546449
Pycard	0.94850844	0.31653556	0.2420692	0.8535377	-0.3919385	0.7044471
Sugt1	0.09159173	0.9126346	0.1262393	0.848884	-0.0975446	0.9137739
Txn1	0.1465255	0.778751	-0.0745583	0.937866	-0.0020518	0.9997147
Txnip	-0.0101596	0.9941421	-0.0766517	0.946901	-0.2102992	0.34853195

Supplementary Figure 10. NLRP3 inflammasome transcriptional gene set analysis for infected mice under CD or KD. In red: $p < 0.05$ (n=3).
