# Supplementary material for: A ketogenic diet can mitigate SARS-CoV-2 induced systemic reprogramming and inflammation
Source: Commun Biol. 2023 Nov 3;6:1115. doi: 10.1038/s42003-023-05478-7 (PMC10624922; doi:10.1038/s42003-023-05478-7)
Supplement: Supplementary file 2 — Description of Additional Supplementary Files [file 42003_2023_5478_MOESM2_ESM.pdf]

Description of Additional Supplementary Files

File name: Supplementary Data 1
Description: GO process enrichment analysis of shared differentially expressed genes (DEGs) due to the exposure to a KD in uninfected animals (KD vs CD), with those changing because of SARS-CoV-2 infection under CD (CD-SARS-CoV-2 vs CD).

File name: Supplementary Data 2
Description: Comparison of differentially expressed genes (DEGs) due to the exposure to a KD in uninfected animals (KD vs CD), with those changing because of SARS-CoV-2 infection under CD (CD-SARS-CoV-2 vs CD).

File name: Supplementary Data 3
Description: Transcriptional regulatory network analysis of RNA-sequencing data (first 500 DEGs ranked according to adjusted significance p value) from mice liver, heart, and kidney, after 7 days from infection with SARS-CoV2, constructed using the TRRUST v2 dataset.

File name: Supplementary Data 4
Description: Transcriptional changes in animals at the endpoint of our study, i.e. mice infected under KD vs CD (KD-SARS-CoV-2 vs CD-SARS-CoV-2).

File name: Supplementary Data 5
Description: DEGs in the liver of uninfected mice because of the KD.

File name: Supplementary Data 6
Description: Network analysis of GO process enrichment in the heart of mice infected under a KD diet.

File name: Supplementary Data 7
Description: Positive and negative metabolite intra- and inter-organ correlations under CD and KD and during infection with SARS-CoV2.

File name: Supplementary Data 8
Description: Metabolite abbreviations.

File name: Supplementary Data 9
Description: Source Data

File name: Supplementary Video 1
Description: Video of mice infected under chow diet.

File name: Supplementary Video 2
Description: Video of mice infected under ketogenic diet.
